# X-ray studies bridge the molecular and macro length scales during the emergence of CoO assemblies

**DOI:** 10.1038/s41467-021-24557-z

**Published:** 2021-07-20

**Authors:** Lukas Grote, Cecilia A. Zito, Kilian Frank, Ann-Christin Dippel, Patrick Reisbeck, Krzysztof Pitala, Kristina O. Kvashnina, Stephen Bauters, Blanka Detlefs, Oleh Ivashko, Pallavi Pandit, Matthias Rebber, Sani Y. Harouna-Mayer, Bert Nickel, Dorota Koziej

**Affiliations:** 1 University of Hamburg, Institute for Nanostructure and Solid-State Physics, Center for Hybrid Nanostructures, Hamburg, Germany; 2 Deutsches Elektronen-Synchrotron DESY, Hamburg, Germany; 3 São Paulo State University UNESP, São José do Rio Preto, Brazil; 4 Ludwig-Maximilians-Universität München, Faculty of Physics and Center for NanoScience (CeNS), Munich, Germany; 5 AGH, University of Science and Technology, Faculty of Physics and Applied Computer Science, Krakow, Poland; 6 Academic Center for Materials and Nanotechnology, AGH University of Science and Technology, Krakow, Poland; 7 The Rossendorf Beamline at the European Synchrotron Radiation Facility ESRF, Grenoble, France; 8 Helmholtz-Zentrum Dresden-Rossendorf (HZDR), Institute of Resource Ecology, Dresden, Germany; 9 European Synchrotron Radiation Facility ESRF, Grenoble, France; 10 The Hamburg Centre for Ultrafast Imaging, Hamburg, Germany

**Keywords:** Structural properties, Characterization and analytical techniques

## Abstract

The key to fabricating complex, hierarchical materials is the control of chemical reactions at various length scales. To this end, the classical model of nucleation and growth fails to provide sufficient information. Here, we illustrate how modern X-ray spectroscopic and scattering in situ studies bridge the molecular- and macro- length scales for assemblies of polyhedrally shaped CoO nanocrystals. Utilizing high energy-resolution fluorescence-detected X-ray absorption spectroscopy, we directly access the molecular level of the nanomaterial synthesis. We reveal that initially Co(acac)_3_ rapidly reduces to square-planar Co(acac)_2_ and coordinates to two solvent molecules. Combining atomic pair distribution functions and small-angle X-ray scattering we observe that, unlike a classical nucleation and growth mechanism, nuclei as small as 2 nm assemble into superstructures of 20 nm. The individual nanoparticles and assemblies continue growing at a similar pace. The final spherical assemblies are smaller than 100 nm, while the nanoparticles reach a size of 6 nm and adopt various polyhedral, edgy shapes. Our work thus provides a comprehensive perspective on the emergence of nano-assemblies in solution.

## Abstract (web summary)

The understanding of nucleation and growth of nanostructures plays a key role in complex materials design. Here, the authors illustrate how X-ray in situ studies link transformation at the molecular- and macro- length scales during the emergence of cobalt oxide assemblies.

## Introduction

**Fig. 1:**
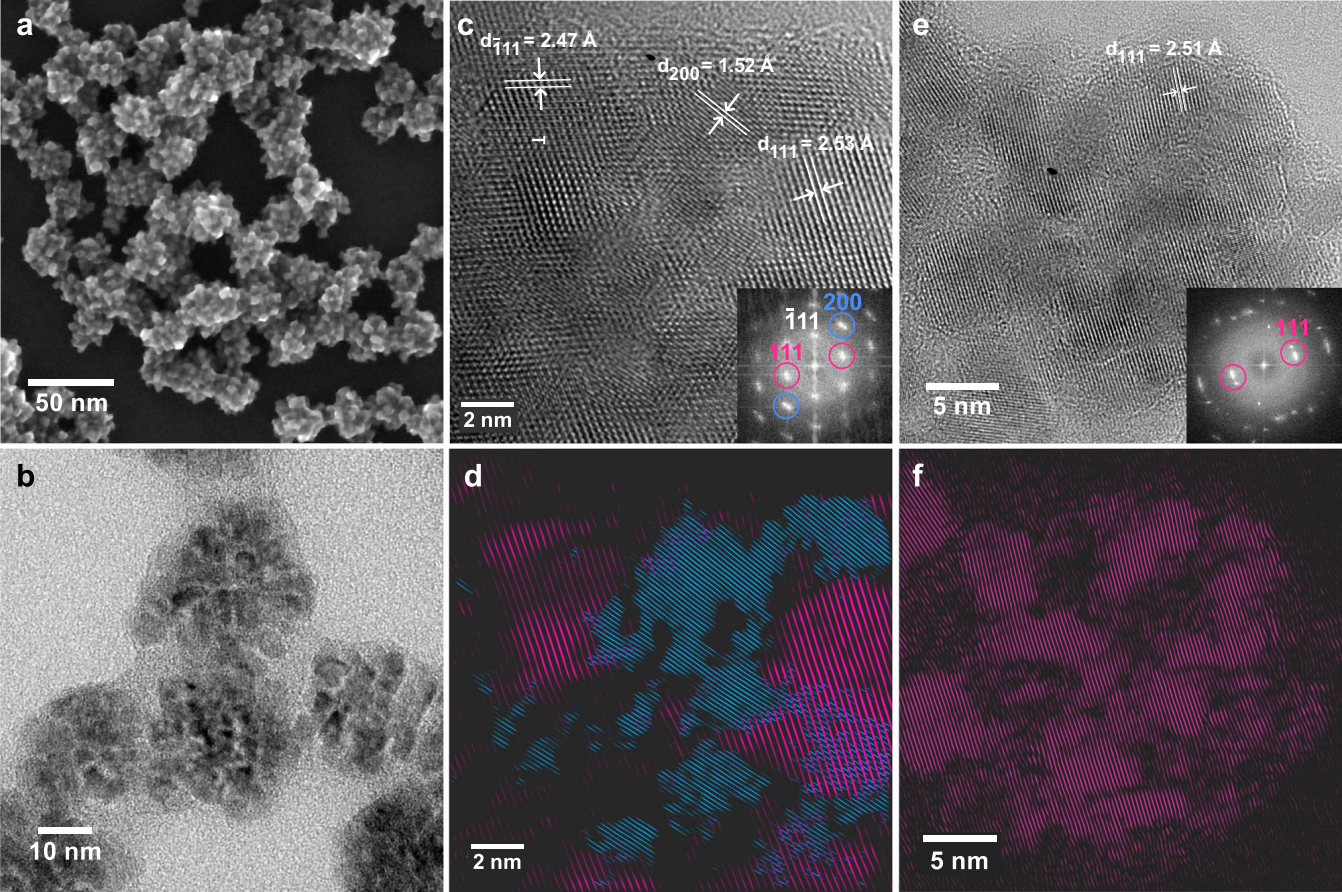
SEM and TEM images of CoO assemblies at the end of the reaction. Different magnifications highlight their hierarchical structure. **a** Probe-corrected SEM image of spherical assemblies built of irregularly shaped crystallites. **b** TEM image of assemblies grown after 90 min of reaction time. **c** HR-TEM image and the corresponding Fourier transformation (FT) (inset) reveal that an assembly exhibits different crystallite orientations. A dislocation in the ($\overline{1}11$) plane is marked with a white T. **d** Filtered image of **c** by inverse FT of the frequency components highlighted by circles in **c** selectively showing the (111) orientation in violet and the (200) orientation in blue. **e** HR-TEM image of a different assembly and its corresponding FT, and **f** filtered inverse FT of **e** highlighting only the (111) orientation. Individual crystallites do not align to each other in a common crystallographic orientation.

With the emerging demand for materials with complex morphological and structural properties, understanding their formation in solution continues to be a major challenge. Most syntheses of colloidal nanoparticles rely on classical crystallization and growth by monomer addition from supersaturated media, which enables the rational synthesis of nanocrystals^1–7^. The recently discovered nonclassical formation pathways involving the assembly of building blocks into distinctive superstructures have opened up completely new ways of complex nanomaterial design^8–11^. Different mechanisms exist within the scope of these nonclassical pathways^10,12,13^. Besides the oriented assembly of nanocrystals into mesocrystals^8,14,15^, the formation of polycrystalline, unoriented assemblies from distinct primary structures^9,16–20^ is a major yet rather unexplored branch (see Cölfen et al. 2019^10^, Fig. 1d). To date, nonclassical nucleation is still far from reaching the level of control established for classical growth pathways. This is by no means a surprise, since the fundamental phenomena cannot be easily generalized, and the existing theory merely provides an overview of possible pathways^10,21,22^. The major experimental difficulties to provide a unique description arise from the fact that we need to study the complex chemical and structural changes on multiple length scales.

To date, synchrotron-based methods such as X-ray absorption spectroscopy and powder X-ray diffraction (PXRD)^23–31^ are routinely combined to study colloidal reactions. However, to follow chemical and electronic changes in complex reactions in solution with high sensitivity and at low concentrations^32–35^, more advanced spectroscopic techniques, such as high energy-resolution fluorescence-detected X-ray absorption near edge structure (HERFD-XANES) are needed. Compared with conventional X-ray absorption spectroscopy, the higher energy resolution of HERFD-XANES makes it sensitive to subtle changes in the local chemical environment of the absorbing atom.

Complementary structural information can be obtained from the pair distribution function (PDF) analysis of high-energy in situ X-ray total scattering. This method takes into account both the Bragg scattering from crystalline and the diffuse scattering from amorphous phases. It results in a real-space representation of the interatomic distances of all constituents in a reaction mixture. In this manner, local ordering during the formation of nuclei, the initial growth of nanocrystals, as well as the restructuring of solvent molecules at the surface of nanoparticles were revealed^36–39^. Self-assembly of building blocks into superstructures can be probed with small-angle X-ray scattering (SAXS)^40–42^. This way, length scales all the way from atomic to macroscopic are accessible via X-ray techniques.

Here, we uniquely combine HERFD-XANES, PDF, and SAXS to study the emergence of CoO nanoassemblies, a noticeable example for a nonclassically crystallized nanostructure. In general, cobalt oxide has applications in lithium ion^43,44^ and lithium oxygen batteries^45^, as a catalyst for electrochemical^46,47^ and for solar water splitting^48–52^. Cobalt precursors under solvothermal conditions^53^ show a complex redox behavior that can lead to CoO, but also to Co_3_O_4_, and to metallic Co particles. Interestingly, the crystal structure of individual CoO nanoparticles determines the growth mechanism. CoO nanoparticles with rock-salt structure have the tendency to form polycrystalline assemblies, while wurtzite CoO preferentially grows into single crystals^21,51,54^. The specific mechanism in solution, however, favoring crystallization into one or the other phase, and the interdependence of crystallite growth and its assembly into superstructures, remains unsolved. Here, starting from Co(III) acetylacetonate (acac), we synthesize phase-pure CoO assemblies with an average size of 58 nm composed out of smaller, polyhedrally shaped, edgy 5–7-nm large particles. We show that HERFD-XANES enables us to track the rearrangement of the organometallic precursor complex accompanying the initial reduction of Co^3+^ to Co^2+^ in solution. PDF reveals the transition from the dissolved Co^2+^ complex to rock-salt CoO nanocrystals by monitoring the changes in bond length between the metal ion and the surrounding oxygen atoms. The combination of PDF and SAXS finally elucidates the interdependence of crystallite growth and assembly that results in the final morphology.

## Results

The reaction of Co(acac)_3_ with benzyl alcohol (BnOH) yields spherical CoO assemblies. They are composed of polyhedrally shaped, edgy nanocrystallites as shown in scanning electron microscopy (SEM) and high-resolution transmission electron microscopy (HR-TEM) images in Fig. 1. From TEM images recorded at different reaction times, we estimate that both the smaller crystallites and the assemblies grow with reaction time (Supplementary Fig. 2–4). Distinct particles are first visible in TEM after 20 min (Supplementary Fig. 5). The crystallites reach an average size of 6 nm and the assemblies have a final size between 20 and 45 nm. The HR-TEM image of a single assembly in Fig. 1c reveals an arbitrary orientation of the crystallites, since different crystallographic planes are exhibited within the same assembly. This can be more easily seen in Fig. 1d and f where we Fourier-filter the HR-TEM image of a single assembly, highlighting crystallites exhibiting the (111) planes in violet and those exhibiting the (200) planes in blue. The assembly of polyhedrally shaped particles into spherical structures implies a nonclassical multistep reaction pathway. To access the complexity of the reaction mechanism, we utilize complementary in situ X-ray spectroscopic and scattering methods.

### Tracking chemical changes of the reactants via in situ X-ray absorption spectroscopy

**Fig. 2:**
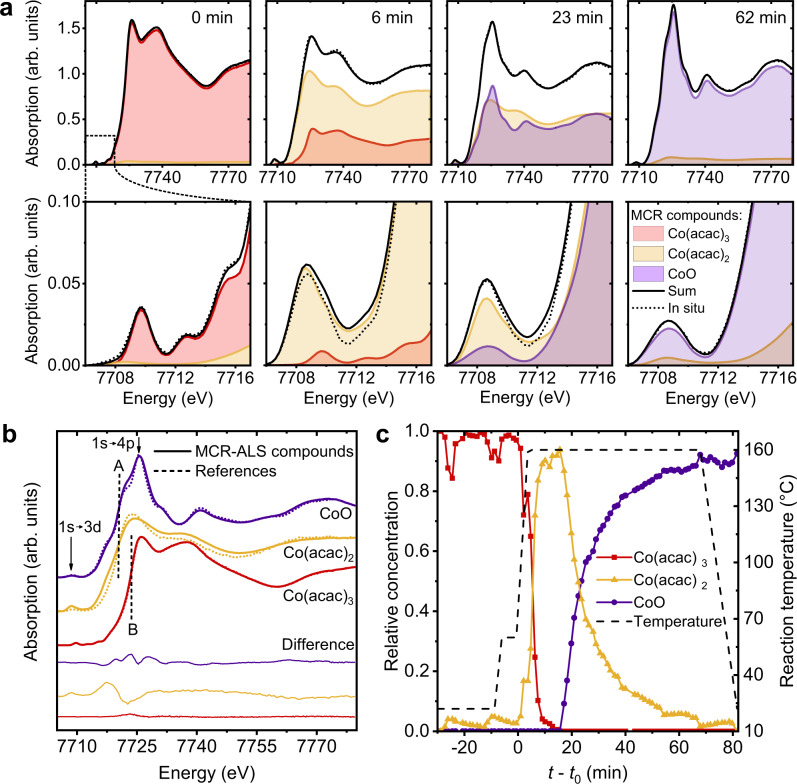
Determination of chemical constituents from the in situ HERFD-XANES study and the MCR-ALS analysis. **a** Selected HERFD-XANES spectra of the time series with the main edge shown in the first and the pre-edge in the second row. The spectra are plotted together with the multivariate curve resolution by alternating least squares (MCR-ALS) compounds recovered at the respective times. Linear combinations of the recovered spectra (black solid lines) match the in situ data (dotted lines). **b** Spectra of the recovered MCR-ALS compounds compared with measured reference spectra of Co(III) acetylacetonate (acac) dissolved in benzyl alcohol (red), Co(acac)_2_ (orange), and CoO (violet). Dashed vertical lines emphasize the edge positions of Co^2+^ (“A” at 7720.2 eV) and Co^3+^ (“B” at 7723.6 eV). **c** Relative concentrations of the MCR-ALS compounds over reaction time. The dashed line indicates the temperature set point. We set *t*_0_ (beginning of reaction time) to the point when we start heating to 160 °C.

To elucidate the chemical reactions in solution, we recorded in situ HERFD-XANES spectra of the CoO assembly synthesis at 160 °C. The full time series is shown in Supplementary Fig. 6. There we notice that, immediately after the reaction temperature is reached, the absorption edge shifts by 3.4 eV toward lower energies, indicating the reduction of the Co^3+^ precursor. Figure 2a depicts four representative stages of the time series in more detail. The multiple features in the pre-edge (second row in Fig. 2a) at 7709.5, 7712.5, and 7716 eV that are present at the beginning of the reaction, reduce to a single feature at 7708.5 eV. These pre-edge features originate from hybridization of 3d and 4p unoccupied states in Co^2+^ and Co^3+^, allowing quadrupole transitions of a 1s core electron into unoccupied 3d states^55–57^. Toward the end of the reaction, we observe an increase of the intensity of the white line at 7726 eV that corresponds to 1s → 4p transitions in CoO, confirming the formation of the final product^56–58^.

We use a multivariate curve resolution by alternating least squares (MCR-ALS) analysis to recover the number and spectra of independent compounds in the reaction mixture and follow changes of their concentration over time^59,60^. This way, we determine the presence of three independent compounds in the in situ data. Further details on the MCR-ALS analysis are given in Supplementary Notes 1 and Supplementary Fig. 7. By comparing linear combinations of the three recovered spectra with the in situ data at different reaction stages (Fig. 2a), we conclude that these compounds accurately describe both the overall spectral features, including the position of the absorption edge and the post-edge fluctuations as well as the pre-edge region. The residual portion of the experimental data not described by the three compounds is 2.1%.

In Fig. 2b, we compare the recovered compound spectra to references. We find that the compound mostly present at the beginning of the reaction (shown in red) is in very good agreement with the reference spectrum of Co(acac)_3_ dissolved in benzyl alcohol. We determine the edge position at 7723.6 eV (labeled B in Fig. 2b) confirming the valency of the precursor^64,65^. For the other compounds, the edge is observed at 7720.2 eV (labeled A in Fig. 2b), indicating that both are in the oxidation state Co^2+^^66–68^. At the end of the reaction, the predominant compound (violet) reproduces all features of the CoO reference that was measured as a dry powder and corrected for self-absorption^69^ (for details, see Supplementary Notes 2 and Supplementary Fig. 8).

**Fig. 3:**
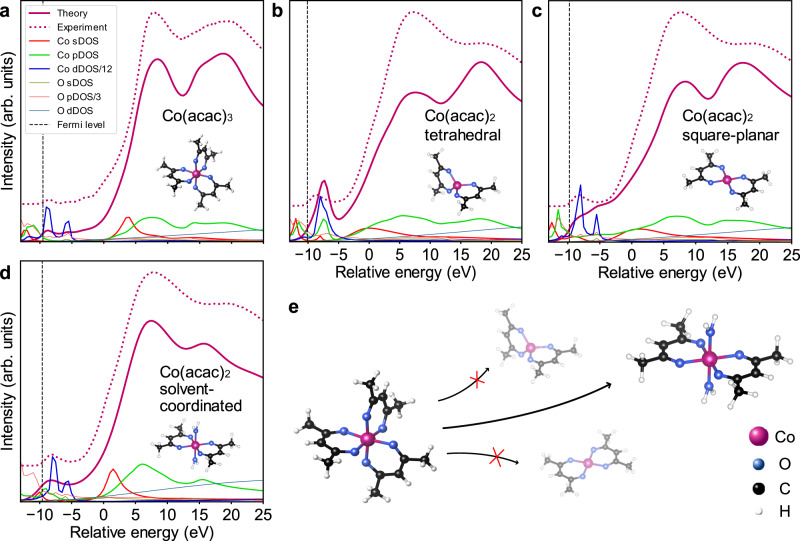
Structure determination of the reaction intermediate Co(acac)_2_. The structure is determined by means of theoretical XANES spectra calculated using the FEFF code^61^. The spectrum of Co(III) acetylacetonate (acac) is shown in **a** for comparison. Examined structures include **b** tetrahedral, **c** square-planar and **d** solvent-coordinated with water as a model solvent, since the precise coordination geometry of benzyl alcohol is unknown. The recovered spectra from the MCR-ALS analysis corresponding to Co(acac)_3_ and Co(acac)_2_ are shown as dotted lines for reference in parts **a** and **b**-**d**, respectively. Spectra are plotted together with the local density of states (DOS) for s, p and d states of cobalt and oxygen atoms. The energy scale is relative to the absorption edge *E*_0_. The position of the Fermi level is indicated for each structure as a vertical dashed line. No instrumental broadening was necessary for the energy resolution of FEFF calculations to be in good agreement with the experimental HERFD-XANES spectra (for details, see Supplementary Notes 4). **e** Structural models of the proposed reaction pathway. The atomic positions in square-planar and tetrahedral Co(acac)_2_ were taken from reference^62^ and the structure of Co(acac)_3_ was adopted from reference^63^.

Most importantly, the compound shown in orange in Fig. 2 compares well to the Co(acac)_2_ reference, which we identify as a reaction intermediate. This is affirmed by the simulation of XANES spectra of Co(acac)_2_ using the FEFF code^61^ as shown in Fig. 3 and Supplementary Fig. 9, 10 (refer to the Supplementary Notes 4 and Supplementary Table 3 for details on the FEFF calculations). Furthermore, FEFF calculations allow deeper insight into the conformation of the reaction intermediate, since the spectral features are very sensitive to the local chemical environment around the absorbing Co^2+^ ion. Tetrahedral and square-planar conformations have been suggested for the isolated Co(acac)_2_ molecule^62^; however, only the calculated XANES spectra of an octahedrally coordinated Co^2+^ ion are in good agreement with the experiment. We thus conclude that in solution, the Co^2+^ ion is additionally coordinated by oxygen atoms of two solvent molecules, forming a bis-adduct of the square-planar Co(acac)_2_ with octahedral coordination^70^. Due to the limited solubility of Co(acac)_2_ in BnOH at room temperature, the reference for the MCR-ALS analysis was measured as a powder where the complex is known to form the tetramer Co_4_(acac)_8_ that also exhibits octahedral coordination^70,71^ (Supplementary Fig. 11d). Thus, we attribute the deviations between the spectrum of the reaction intermediate recovered by MCR-ALS and the Co(acac)_2_ reference in Fig. 2b to slight changes in the coordination geometry and the second-shell chemical environment upon dissolving.

To quantify the portion of each compound in the system, in Fig. 2c, we examine the profiles of the relative concentrations over time that result from the MCR-ALS analysis. When the temperature reaches the set point, BnOH reduces Co(acac)_3_ to Co(acac)_2_ within 10 min, significantly faster than the formation of CoO from Co(acac)_2_. Both reaction steps are best described by pseudo-first-order kinetics^72,73^ (for details, see Supplementary Notes 3 and Supplementary Fig. 9) with the rate of the initial reduction being significantly higher than that of the formation of the final product. We ascribe the fluctuations in the relative concentrations most notable in the beginning and at the end of the reaction to changes of the position of the X-ray beam on the reaction cell, in combination with no stirring applied. This can be a reason why the relative concentration of Co(acac)_2_ never reaches 100%. Precipitation of large particles could be another reason why this is neither the case for CoO. However, it is important to note that the formation of CoO only sets in after the precursor has been completely reduced to Co(acac)_2_. We can thus exclude the possibility of parallel reactions such as the precursor reacting to CoO without a stable intermediate.

### Pinning down nucleation and growth of nanoparticles with in situ PDF

X-ray spectroscopy provides information about the chemical state of cobalt species. However, it fails to investigate the crystallization and growth of the CoO nanoparticles from a structural perspective. Therefore, we performed in situ total X-ray scattering experiments of the synthesis at 160 °C. Supplementary Fig. 12 shows the integrated and background-subtracted in situ total X-ray scattering data. The constant broad feature at around 1 Å^−1^ results from the remaining scattering from the sample environment (glass vessel filled with BnOH) after background subtraction (Supplementary Fig. 13). Except for the residual background, no reflections are seen either in the initial stages of the reaction or during the step of heating. The crystallization of CoO starts after ca. 20 min of reaction time suggested by the appearance of CoO reflections, which persist until the reaction finishes after 90 min.

**Fig. 4:**
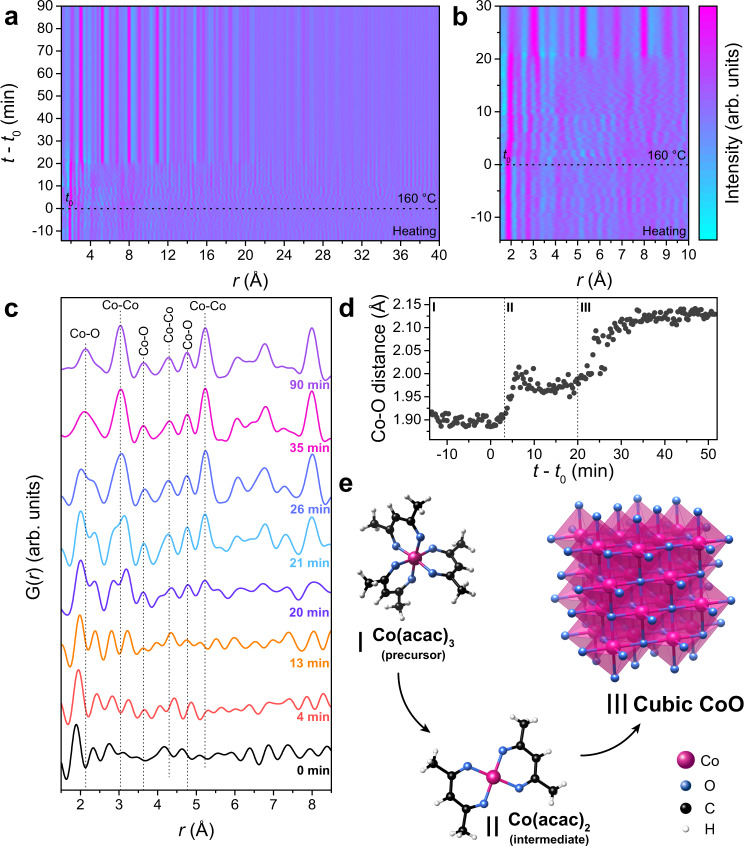
Following the structural rearrangement of the Co complex and the crystallization of CoO. **a** In situ time-resolved PDFs G(*r*) for the CoO synthesis. The heating step includes heating from room temperature to 60 °C, 5 min at 60 °C, and heating from 60 to 160 °C. We set *t*_0_ (beginning of reaction time) to the point when we start heating to 160 °C. **b** Region of **a** illustrating the short-range order before the crystallization of CoO. **c** Zoom into the local order region of the PDFs for selected reaction times of the in situ data. Dashed lines show the first coordination shells of CoO. **d** Changes in the shortest Co–O bond over the reaction course, which indicate three reaction stages. **e** Structural models for the proposed reaction stages. The structures were obtained from the crystallographic data from references^63,74,75^ using the VESTA software^76^.

Total scattering data encode structural information on chemical constituents independent of their aggregation state and ordering, and thus enable to study precursor molecules in solution, as well as amorphous and crystalline nanoparticles. To access this information, we convert the in situ-recorded scattering data into the time-resolved PDFs (G(*r*)), as shown in Fig. 4a. We obtain a real-space representation of the interatomic distance changes of all compounds in the reaction mixture over the course of the reaction. At the beginning, the time-resolved PDFs reveal only a short-range order within a correlation length *r* of less than 5 Å, indicating the presence of isolated molecular species. The long-range order suddenly appears after 20 min, pinning down the nucleation of the crystalline CoO phase.

The low *r* region of the time-resolved PDFs before the crystallization as seen in Fig. 4b clearly shows a single and intense feature at *r* ca. 2 Å that is constant until longer-range order appears. This peak represents the nearest-neighbor Co–O bond. However, the position of this peak starts shifting to higher *r* values only 4 min after heating to the final reaction temperature is started, revealing the initial changes of the Co environment in the precursor prior to nucleation. To quantify this behavior, the PDFs corresponding to the selected reaction stages are depicted in Fig. 4c. At early reaction times below 13 min, the PDFs show only local order of the reactant. As the reaction progresses further to 20 min and beyond, the peaks related to the structure of CoO first on the short-to-medium range and soon also on the long-range emerge and intensify as indicated by the dashed lines. Moreover, small features at around 2.34, 2.76, and 3.14 Å in the initial stage match very well with the C–O and two different Co–C bond distances in the Co(acac)_3_ molecule^63^. These peaks change position over time and disappear when the crystalline CoO phase appears, most noticeably when the peak of the nearest Co–Co distance at around 3.01 Å emerges. The overall behavior is similar to ex situ reference measurements after selected reaction times, as shown in Supplementary Fig. 14a,b.

The observed shifts of PDF peaks during the reaction suggest that the effective Co–O distance can act as a fingerprint of the nucleation pathway. To test this approach, we depict the changes in the shortest Co–O distance over reaction time in Fig. 4d. Simple eye inspection reveals three stages for the reaction. At room temperature and during heating, the Co–O distance remains close to ~1.90 Å (stage 1), which agrees well with the bond length obtained from our ex situ measurements of the Co(acac)_3_ reference (Supplementary Fig. 14c). After about 4 min, the Co–O bond length rapidly increases and fluctuates around 1.98 Å until 20 min, suggesting an intermediate state (stage 2). Then, the Co–O distance gradually expands further up to 30 min, where it approaches a value of ~2.12 Å that remains close to constant (stage 3). It is worth noting that the transformation from stage 1 to 2 occurs much faster than the final changes from stage 2 to 3, matching the reaction rates found by the HERFD-XANES study (Fig. 2c).

The reaction stages can thus be explained as follows. Stage 1 is associated with the precursor Co(acac)_3_ without noticeable changes. For stage 2, HERFD-XANES pointed out Co(acac)_2_ as the reaction intermediate at comparable reaction times as those inferred from Fig. 4d. Thus, it is reasonable that the Co–O bond lengthening associated with the lack of long-range order corresponds to the reduction of the precursor to Co(acac)_2_ during stage 2, although the ex situ measurement of the Co(acac)_2_ reference at room temperature provides a slightly longer Co–O bond length of 2.05 Å (Supplementary Fig. 14c). Finally, stage 3 corresponds to the formation of CoO nanoparticles, as the Co–O distance approaches that of the first coordination shell in rock-salt CoO. The proposed reaction course is illustrated in Fig. 4e.

**Fig. 5:**
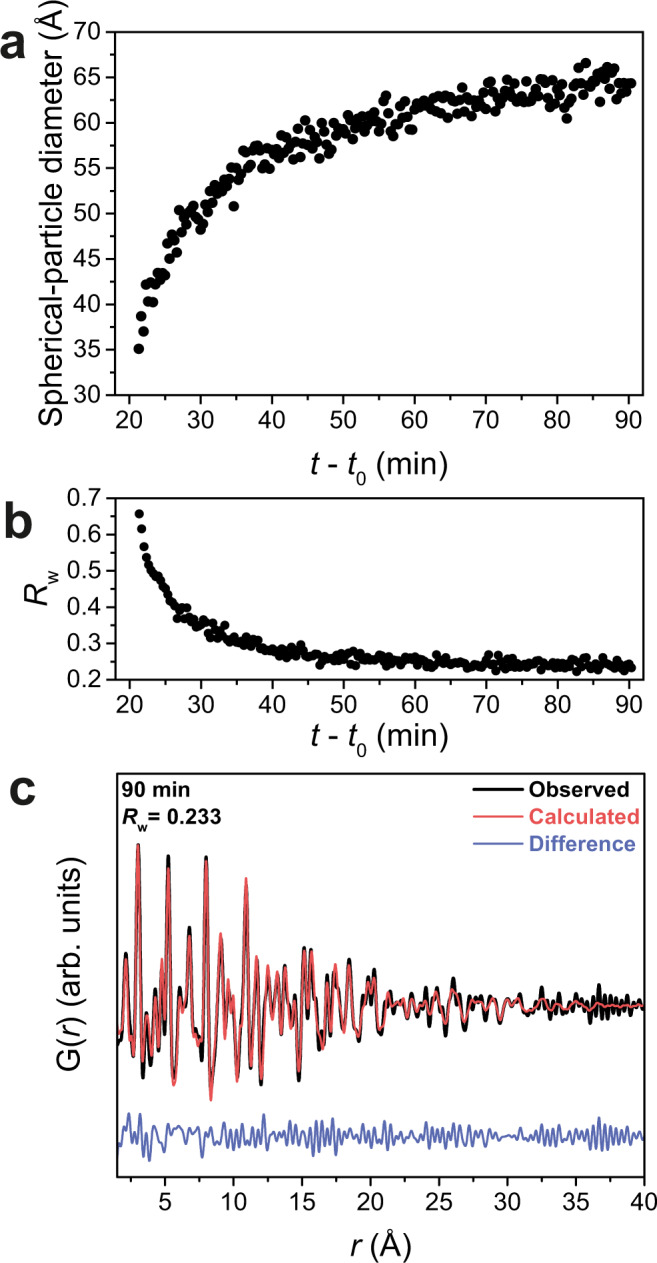
Results of the sequential PDF refinement. **a** Evolution of sp diameter as a function of the reaction time. **b** Fit-quality *R*_w_ progression as a function of the reaction time. The data in **a** and **b** were obtained from the sequential refinement of time-resolved PDFs. **c** Experimental and calculated PDFs G(*r*) for the reaction at 160 °C after 90 min. The calculation imposes a rock-salt CoO phase^75^.

To track the structural evolution of the CoO particles in the final phase 3, we perform a sequential refinement on the time-resolved PDFs starting from the final product after 90 min, and trace the signal back toward earlier reaction times. In this process, calculated PDFs are fitted to the data in the *r* range between 1.5 and 40 Å applying the crystal structure of cubic CoO. Besides other structural parameters, we refine the spherical particle (sp) diameter that represents the size of individual crystallites in PDF. Figure 5a shows the sp diameter as a function of the reaction time. The refinement results for the other parameters are depicted in Supplementary Fig. 15. At 21 min 20 s, the initial value of the sp-diameter is ~35 Å, increasing rapidly to ca. 50 Å within additional 10 min. Crystallites gradually continue to grow until the end of the reaction at 90 min and reach a diameter of 64 Å. At the final reaction stage, the crystallite sizes are in good agreement with the size of 6 nm of the small building blocks of the assemblies visible in TEM images as shown in Fig. 1. The lattice parameter *a* rapidly decreases as the reaction progresses and fluctuates around 4.28 Å throughout most of the reaction time. It is worth noting that the sequential refinement was also carried out in a higher *r* range of 1.5–60 Å, comparable to the size of the small building blocks, and similar refinement results are observed as illustrated in Supplementary Fig. 16.

The fit-quality *R*_w_ (lower is better) corresponding to the PDF refinement presented in Fig. 5a and Supplementary Figure 15 is shown in Fig. 5b as a function of the reaction time. For the reaction time before 40 min, the co-existence of Co(acac)_2_ and CoO induces a misfit in the low *r* region. The sp diameter is robust against the misfit in this region and thus the relative changes of crystallite size are reliable. However, the CoO structural model does not well describe the experimental data before 40 min as shown in Supplementary Fig. 17a. After 40 min and beyond, the PDF data and fit match well, with *R*_w_ dropping below 0.3, and improving continuously with reaction time. The modeling of the PDF obtained at 90 min reveals good agreement with the cubic CoO structure in the whole *r* range, i.e., a low *R*_w_ of 0.233, as depicted in Fig. 5c. Additional refinements of the PDFs at other reaction stages are shown in Supplementary Fig. 17b–d.

The in situ reaction at a milder temperature of 140 °C compared with the reaction at 160 °C discussed so far presents a similar trend of the formation of the CoO nanoparticles. The kinetics and growth rate are, however, significantly slower than those at 160 °C, as discussed in Supplementary Notes 5 and Supplementary Figs. 18, 19.

### Tracking the assembly with SAXS

**Fig. 6:**
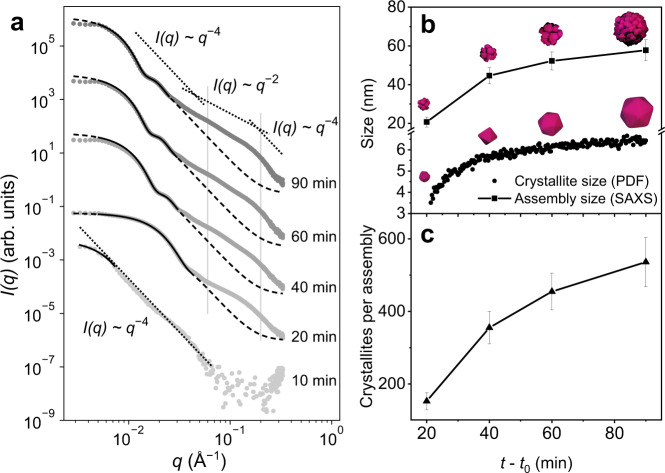
SAXS data modeling to follow the assembly of CoO nanocrystals. **a** Small-angle X-ray scattering (SAXS) intensities *I*(*q*) of CoO assemblies for different reaction times. Dashed curves: calculated intensities for homogeneous spherical assemblies. Superimposed bold curves indicate the least-squares fitting range. Dotted lines: power laws describing the contributions of compact particle shape (*q*^−4^) and corrugated surface (*q*^−^^2^) on the different length scales. An intermediate region is emphasized by vertical gray lines. **b** Comparison of size evolution over time of polyhedra (spherical particle diameter obtained from in situ pair distribution functions (PDF)) and assemblies (obtained from SAXS). Here, the error bars indicate the polydispersity of the assemblies. **c** Average number of crystallites per assembly obtained from average assembly size in **b** assuming that they are built from densely packed spherical units with a filled volume fraction of 74%. Here, the error bars result from the error propagation of the average assembly sizes (SAXS) and of the standard deviation of the crystallite sizes (PDF) in **b**.

So far, we have elucidated the reaction pathway from the dissolved precursor to the product phase. While with PDF analysis we have been able to follow nucleation and growth, information about the process of crystallite assembly into the superstructure is missing. The size and the internal structure of assemblies in solution are accessible from the scattered X-ray intensity at small scattering angles 2*θ*, here below 4.2°. We show this SAXS data as a function of scattering vector $q=\frac{4\pi }{\lambda }\,\sin (\theta )$, where *λ* is the X-ray wavelength. SAXS intensities *I*(*q*), shown in Fig. 6a, were recorded ex situ from aliquots obtained by ceasing the reaction at five different time points. After 10 min of reaction time, the SAXS intensity shows a pronounced divergence at low *q*. The intensity obeys approximately a* q*^*−*4^ power law characteristic for scattering contrast from objects with well-defined boundaries^77,78^. The slight kink below *q* = 0.01 Å^−1^ indicates that the characteristic length scale of the scattering objects (2π/*q*) is a few 10 nm. At this reaction stage, the intermediate Co(acac)_2_ dominates the reaction mixture according to our HERFD-XANES measurements reported in Fig. 2c. Since the intermediate is not well soluble in BnOH at room temperature, the scattering signal visible in SAXS at this stage may be from precipitates of the intermediate.

From 20 min reaction time onward, the SAXS signal at low angles, i.e., low values of *q*, is well described by an intensity plateau region and a subsequent intensity drop. This is clear evidence that well-defined objects have formed. A fit with a model assuming homogeneous spherical assemblies (solid line in Fig. 6a, see also Supplementary Notes 6 and Supplementary Table 4) yields an assembly diameter of *D* = 21 nm. Additionally, a size polydispersity of *ΔD/D* = 25% is obtained. For 40, 60, and 90 min reaction time, the assembly diameter gradually increases, i.e., the plateau region narrows. The sphere size obtained from the analysis levels off at a diameter of *D* = 58 nm, *ΔD/D* = 19%, as summarized in Fig. 6b.

For larger diameters, a second intensity oscillation is observed in the data close to the plateau region, as expected for scattering from homogeneous spheres. The homogeneous spherical model, however, only describes the SAXS intensity well up to *q* = 0.04 Å^−^^1^. For larger *q* values, the model strongly underestimates the SAXS intensity, indicating a more complex internal structure.

Particles with pronounced internal structure, such as fractal and porous materials, are known to show a crossover of the power law underlying the SAXS intensity, at the point where *q* is large enough to resolve these internal structures^77^. Our data suggest that such a crossover from initially *q*^−^^4^ to *q*^−^^2^ occurs here at ca. *q* = 0.06 Å^−1^, i.e., for length scales smaller than 10 nm, illustrated by the dotted lines in Fig. 6a. The region described by *q*^−^^2^ extends up to *q* = 0.2 Å^−1^ (indicated by vertical lines) beyond which the *q*^−4^ behavior is recovered. Thus, the scattering signal in the intermediate region originates from inhomogeneities at length scales of ca. 3 nm up to 10 nm. This size range is considerably smaller than the full diameter of the assemblies but coincides with the size range of the building units determined with PDF (sp diameter in Fig. 5a). On an even smaller scale, i.e., for *q* > 0.2 Å^−1^, the assemblies scatter like compact objects. In SAXS, the dimensional crossover is seen for all reaction times between 20 and 90 min, suggesting that the assemblies are corrugated and porous throughout the reaction. The SEM and TEM images shown in Fig. 1 and Supplementary Figs. 2, 3 and 22 also display that the compact crystallites form porous assemblies with voids in the interior.

We measured samples after selected reaction times in our laboratory SAXS system. Here, we are able to calibrate the SAXS data on absolute scale, allowing us to determine the volume fraction of the reactant (for details, see Supplementary Notes 6). At the end of the reaction, the volume fraction of the assembly is 0.026%, corresponding to a CoO concentration of 1.65 mg/ml and a reaction yield of ca. 22%. However, since these samples were measured ex situ, precipitation may have led to an underestimation of the concentration and thus the reaction yield. The final assembly size of *D* = 58 nm reported by SAXS is in good agreement with TEM images taken from the same batch used for SAXS analysis (Supplementary Fig. 22). However, assemblies are about 30% larger than those from another batch shown in TEM images of Fig. 1. Even though the absolute values vary between the batches, we observed the similar growth trends independent of the batch. We measured PXRD for the same samples as used for the SAXS analysis, yielding cubic CoO domain sizes of 4.9 ± 2.8 nm, 5.8 ± 1.0 nm, and 6.0 ± 1.2 nm by Scherrer analysis for 40, 60, and 90 min reaction times, respectively. For details, refer to Supplementary Table 5 and Supplementary Fig. 21. These values are in good agreement with the crystallite sizes found by the in situ PDF analysis.

The average number of crystallites per assembly can now be estimated as a function of reaction time. For this purpose, we compare the assembly size as obtained from SAXS with the diameter of the crystallites, i.e., the sp diameter as obtained from the PDF refinement (Fig. 6b). We plot the resulting number of crystallites per assembly in Fig. 6c, assuming dense packing of spherical units with a filled volume fraction of 74%. From the similarity of the three curves, we conclude that during the entire evolution of the CoO phase, both the attachment of crystallites onto the assemblies and the growth of the individual crystallites contribute similarly to the resulting growth of the assemblies. Moreover, time-resolved TEM images shown in Supplementary Fig. 2 and 3 support our findings and show that during all reaction stages, crystallites in the center of the assemblies are of the same size as the ones at the surface.

## Discussion

**Fig. 7:**
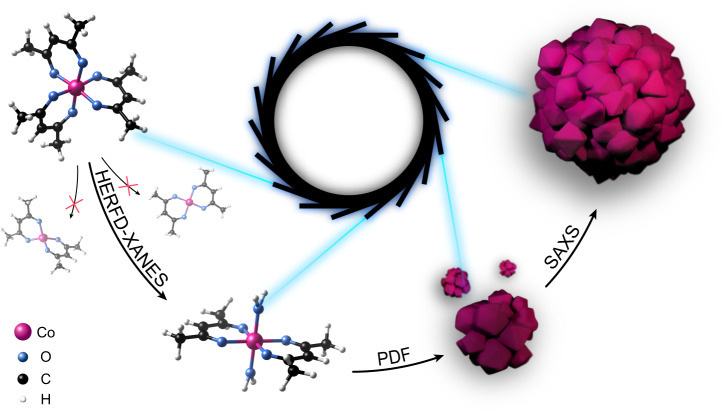
Overview of the emergence of nano-assemblies of CoO in benzyl alcohol. Complementary X-ray spectroscopic and scattering methods enable the bridging of the studies of the reaction mechanism from molecular- to macro- length scales. We combined high energy-resolution fluorescence-detected X-ray absorption near edge structure (HERFD-XANES), pair distribution function (PDF) analysis and small-angle X-ray scattering (SAXS) to follow the emergence of CoO nanoassemblies in solution.

Previous studies of nonclassical crystallization mechanisms suggest that multiple pathways exist involving distinct precrystalline entities (see Cölfen et al. 2019^10^, Fig. 1d therein and references). Our work provides an experimental complement to this work. Using the example of polycrystalline assemblies of polyhedrally shaped CoO nanoparticles, we illustrate that advanced X-ray spectroscopic and scattering in situ studies are very powerful tools for bridging the molecular- and macro length scales. Putting together complementary pieces of information, we can pin down the formation of monomers, nucleation, growth, and assembly of the CoO polyhedra as schematically shown in Fig. 7 and Supplementary Fig. 23. In summary, HERFD-XANES revealed Co(acac)_2_ as a monomer species that quickly forms upon reduction of the precursor in solution. FEFF calculations indicated that the intermediate forms a bis-adduct of square-planar Co(acac)_2_ with coordinated solvent molecules. Total scattering and PDF analysis gave insights into local structural changes around the Co^2+^ ion during nucleation and growth of the cubic CoO phase. We assigned the stepwise increase of the Co–O bond length to the rearrangement of the organo-metallic precursor upon reduction, and to the nucleation of the crystalline product phase. SAXS measurements together with crystallite-size evolution derived from PDF shed light on the assembly mechanism of the final structure. They indicate that crystallites start assembling soon after nucleation and continue to assemble, while the cavernous nature of the assemblies allows crystallites to grow similarly at the surface and in bulk. During the entire reaction, growth and assembly of particles contribute to the size evolution of the final product. The size evolution of crystallites and assemblies, as well as the average number of crystallites per assembly, follows the kinetics of the formation of the product phase. Our findings do not support a classical crystallization and growth mechanism, in which crystallites nucleate and grow only at the surface of the assemblies, until they are covered with the next layer of crystallites. On the contrary, the cavernous structure of the nanoparticle assemblies allows the crystallites to always consume material from the solution and thus to grow both on the surface and in bulk. In addition, no isolated, unassembled crystallites are found in SAXS or TEM, indicating that crystallites, which may nucleate in solution, are soon attached to an assembly, keeping the number of isolated crystallites low.

The simultaneous growth and assembly of crystallites we observe here is a markedly different mechanism from, e.g., evaporation-induced assembly of pre-formed nanocrystals in dispersion to mesocrystals, which has been in the focus of recent in situ investigations by X-ray methods^79–81^. Here we find that for the synthesis of CoO nanocrystals under solvothermal conditions, crystallite nucleation and growth are accompanied by unoriented assembly into a polycrystalline superstructure.

More generally, our approach of combining three techniques from the fields of X-ray spectroscopy and scattering made it possible to gain mechanistic insights into all steps of complex nonclassical crystallization. While spectroscopy is focused on the chemistry, scattering in the full range of reciprocal space is essential to follow the structural and morphological evolution at different length scales, both key features when it comes to understanding the complexity of nanomaterial formation.

We demonstrate a way to strengthen the use of multimodal in situ X-ray experiments, in particular, for studying nonclassical crystallization processes. The level of microscopic detail obtained within this study encourages the application of this approach in related fields, which might benefit even more from the combination of structural and spectroscopic synchrotron techniques. Particularly, catalysts at functional interfaces, which are notoriously difficult to probe in a coherent chemical and structural way, might be worthwhile to explore^82^.

## Methods

### Synthetic procedure

Chemicals: Benzyl alcohol (>99%), Co(acac)_3_ (99.99%), and Co(acac)_2_ (>99%) were purchased from Sigma-Aldrich, and ethanol (absolute) for washing from VWR. All chemicals were used without further purification.

Synthesis: Co(acac)_3_ (0.5 mmol) was added to 5 mL of BnOH and stirred for 5 min at room temperature. A quantity of 0.8 mL of the green solution was transferred to the reaction container of the in situ cell (Supplementary Fig. 1), sealed, and heated at an average heating rate of 1 °C/s first to 60 °C for 5 min to homogenize, then at the same rate to 140 °C or 160 °C, and stabilized at this temperature during 210 min or 90 min, respectively, for in situ experiments. We define the beginning of the reaction time (*t*_0_) at the point when the heating to the reaction temperature of 140 °C or 160 °C starts. All mentions of reaction time are relative to *t*_0_. For ex situ SAXS measurements, the reaction was stopped after 20, 40, 60, and 90 min of reaction time by quickly cooling down the in situ cell to room temperature with a cold metal block, and the solution was measured without further treatment. For SEM/TEM, the brown precipitate was washed three times with ethanol and dried at 60 °C. For the TEM image in Supplementary Fig. 22, no washing was applied.

SEM: Scanning electron microscopy images were taken and probe-corrected with a Regulus 8220 (Hitachi High Technologies Corp., Japan) at an acceleration voltage of 10 kV and using the secondary electron signal.

TEM: Transmission electron microscopy images and electron diffraction (ED) measurements were taken with a JEM 1011, high-resolution TEM images with a JEM 2200 FS (JEOL Ltd., Japan) at an acceleration voltage of 100 kV.

### In situ reactor

The reactor and measurement cell (Supplementary Fig. 1) is composed of a PEEK reaction container inserted into a heated brass housing and surrounded by thermally insulating alumina bricks. The container is sealed by a PEEK cap pressed against the top side of the container. A 0.2-mm-thin PEEK wall on one side of the container serves as the entrance and exit window for fluorescence-detected X-ray absorption spectroscopy. For total scattering measurements, a cylindrical quartz capillary with a diameter of 6.5 mm and a wall thickness of 0.5 mm is held in place by a PEEK support not in contact with the primary X-ray beam in order to avoid background signal from the semicrystalline PEEK. No stirring is applied to the reaction solution.

### X-ray techniques

HERFD-XANES: Spectra were recorded at beamline ID26 at the European Synchrotron Radiation Facility (ESRF), Grenoble, France. Using a Si (111) double-crystal monochromator (DCM), the incident energy was varied from 7.70 to 7.78 keV over the Co K edge. The maximum of the Co Kα_1_ (1s2p) fluorescence line was selected with an emission spectrometer in Rowland geometry with five Si (531) analyzer crystals aligned at the Bragg angle of 77°. HERFD-XANES spectra were recorded in continuous scan mode every 80 s with an overall energy resolution of 1.22 eV and average energy steps of 0.05 eV. The position of the beam on the in situ cell was changed after each scan to avoid beam damage. The CoO reference was measured as a dry powder using a Si (311) DCM and the Co(acac)_2_ reference was measured at beamline BM14 at ESRF selecting the Kβ_1,3_ (1s3p) emission line with one Ge (444) analyzer crystal aligned at the Bragg angle of 83°^83^. Spectra were later aligned to the absorption edge to account for possibly different energy calibrations.

Total X-ray scattering: Data were taken at beamline P21.1 of PETRA III at Deutsches Elektronen-Synchrotron (DESY), Hamburg, Germany. Diffraction patterns were recorded every 10 s at an X-ray energy of 102.92 keV (λ = 0.121 Å) using a digital X-ray flat-panel detector XRD1621 (Perkin Elmer Inc., USA) with a pixel size of 200 × 200 μm² and a sample-to-detector distance of 0.411 m, obtained from a calibration with a CeO_2_ powder standard packed into the quartz capillary of the in situ cell.

SAXS: Data were obtained at beamline P03 at PETRA III^84^. The X-ray energy was 12.9 keV (λ = 0.961 Å). Samples were loaded in 2 mm quartz glass capillaries (Hilgenberg, Malsfeld, Germany). Data were recorded with a Pilatus 1 M detector (Dectris, Baden-Dättwil, Switzerland) for 5 × 0.1 s at 5.2 m and for 0.5 s at 2.0 m sample-to-detector distance, and combined. Additional SAXS data were recorded at a sealed tube Mo anode microfocus X-ray setup at LMU, Munich^85^. A Pilatus 300 K detector (Dectris, Baden-Dättwil, Switzerland) was used at 1.0 m sample-to-detector distance. Samples were loaded in custom-made chambers with Kapton foils (DuPont, USA) as windows and measured for 5 × 20 min and 7 × 20 min before taking the median, for the samples after 20 and 90 min of reaction time, respectively.

PXRD: Data were recorded using the laboratory molybdenum anode microfocus X-ray setup at LMU, Munich^85^. The X-ray energy was 17.4 keV (*λ* = 0.71 Å). Samples were dried for three days on Kapton foils (DuPont, USA) for measurements. A Pilatus 100 K detector (Dectris, Baden-Dättwil, Switzerland) was raster-scanned perpendicular to the beam to enlarge the *q* range. The total exposure time per sample was 18 h. A LaB_6_ powder standard was used for calibration of the sample-to-detector distance and the instrumental resolution.

### Data analysis

HERFD-XANES: Data reduction of HERFD-XANES spectra in the energy range from 7.705 to 7.780 keV was performed with the software package PyMCA^86^. Binning was applied to reduce the number of data points by a factor of five and the edge step was normalized by fitting a constant to the pre- and post-edge regions. Edge positions were calculated and spectra were aligned with Athena, part of the Demeter software package^87^. The in situ data set was analyzed with the MCR-ALS method implemented in MATLAB (The MathWorks Inc., USA)^59,60,88^. An uncertainty estimation with a noise level of 3% was applied. The number of components was determined by means of singular value decomposition and the initial spectra and concentration profiles were estimated by the purest variables detection method. The ALS algorithm was applied with the following constraints: (1) non-negativity of spectra and concentrations, (2) unimodality of concentrations with 20% tolerance, and (3) convergence criterion of 0.1.

FEFF: Theoretical XANES spectra were calculated using the FEFF code^61^ in version 9.6.4 with the Hedin–Lundquist energy-dependent exchange correlation potential. The settings are listed in Supplementary Table 3. For the Co_4_(acac)_8_ tetramer, calculations with each of the Co ions set as the absorber were averaged, since their environments are not equivalent. For additional details, refer to Supplementary Notes 4. Experimental and theoretical spectra were aligned with Athena^87^.

Total scattering: The azimuthal integration of the 2D diffraction patterns was performed with pyFAI^89^. CeO_2_ powder packed into the quartz capillary was used as a calibrant for the integration and the instrumental parameters in real space. The integrated data were normalized and further processed using the program xPDFsuite^90^ with PDFgetX3^91^ to perform the background subtraction, normalization to the atomic form factors, and Fourier transformation to obtain the PDF (G(*r*)). For the background subtraction, the glass capillary filled with BnOH was measured under the same conditions as for the synthesis. *Q*_min_ and *Q*_max_ parameters were set to 0.59 Å^−^^1^ and 16.5 Å^−1^, respectively. The resulting PDFs were sequentially refined applying PDFgui^92^. *Q*_damp_ and *Q*_broad_ were determined as 0.0463 and 0.0574 Å^−1^, respectively, from the CeO_2_ calibrant. Structural refinements were performed using crystallographic data of cubic CoO (*Fm-3m* (225) space group) from the Inorganic Crystal Structure Database (ICSD)-9865^75^.

SAXS and PXRD: Intensities were azimuthally averaged and binned using the Nika^93^ package for Igor Pro (Wavemetrics, Portland OR, USA). A solution of Co(acac)_3_ in BnOH was recorded as background and subtracted from all SAXS data of ex situ samples of the synthesis. A Kapton foil background was recorded and subtracted from the PXRD data. For additional details, refer to Supplementary Notes 6 and 7.

## Supplementary information

Supplementary Information

## Data Availability

The SEM, TEM, HERFD-XANES, total X-ray scattering, PXRD and SAXS datasets generated and analyzed during the current study are available in the zenodo repository, 10.5281/zenodo.4746349^94^. Supplementary Figures 1–21, Supplementary Tables 1–5 provide TEM and HR-TEM images, details on MCR-ALS method, HERFD-XANES self-absorption correction, reaction kinetics, FEFF simulations, SAXS modeling and PXRD analysis, additional total scattering and PDF data.
