# Supplementary material for: X-ray studies bridge the molecular and macro length scales during the emergence of CoO assemblies
Source: Nat Commun. 2021 Jul 20;12:4429. doi: 10.1038/s41467-021-24557-z (PMC8292528; doi:10.1038/s41467-021-24557-z)
Supplement: Supplementary file 1 — Supplementary Information [file 41467_2021_24557_MOESM1_ESM.pdf]

X-ray studies bridge the molecular and macro length scales during the emergence of CoO assemblies

Authors: Lukas Grote,[1,2] Cecilia A. Zito,[1,3] Kilian Frank,[4] Ann-Christin Dippel,[2] Patrick Reisbeck,[4] Krzysztof Pitala,[5,6] Kristina O. Kvashnina,[7,8] Stephen Bauters,[7,8] Blanka Detlefs,[9] Oleh Ivashko,[2] Pallavi Pandit,[2] Matthias Rebber,[1,10] Sani Y. Harouna-Mayer,[1,10] Bert Nickel,[4] and Dorota Koziej [1,10,*]

Affiliations:

[1] University of Hamburg, Institute for Nanostructure and Solid-State Physics, Center for Hybrid Nanostructures, Luruper Chaussee 149, 22761 Hamburg, Germany

[2] Deutsches Elektronen-Synchrotron DESY, Notkestraße 85, 22607 Hamburg, Germany

[3] São Paulo State University UNESP, Rua Cristóvão Colombo 2265, 15054000 São José do Rio Preto, Brazil

[4] Ludwig-Maximilians-Universität München, Faculty of Physics and Center for NanoScience (CeNS), Geschwister-Scholl-Platz 1, 80539 Munich, Germany

[5] AGH, University of Science and Technology, Faculty of Physics and Applied Computer Science, Al. Mickiewicza 30, 30-059 Krakow, Poland

[6] Academic Center for Materials and Nanotechnology, AGH University of Science and Technology, Kawiory, 30-055 Krakow, Poland

[7] The Rossendorf Beamline at the European Synchrotron Radiation Facility ESRF, 71 Avenue des Martyrs, 38000 Grenoble, France

[8] Helmholtz-Zentrum Dresden-Rossendorf (HZDR), Institute of Resource Ecology, Bautzner Landstraße 400, 01328 Dresden, Germany

[9] European Synchrotron Radiation Facility ESRF, 71 Avenue des Martyrs, 38043 Grenoble, France

[10] The Hamburg Centre for Ultrafast Imaging, Hamburg, Germany

These authors contributed equally: Lukas Grote, Cecilia A. Zito, Kilian Frank

* Correspondence to: dorota.koziej@physnet.uni-hamburg.de

Supplementary Information

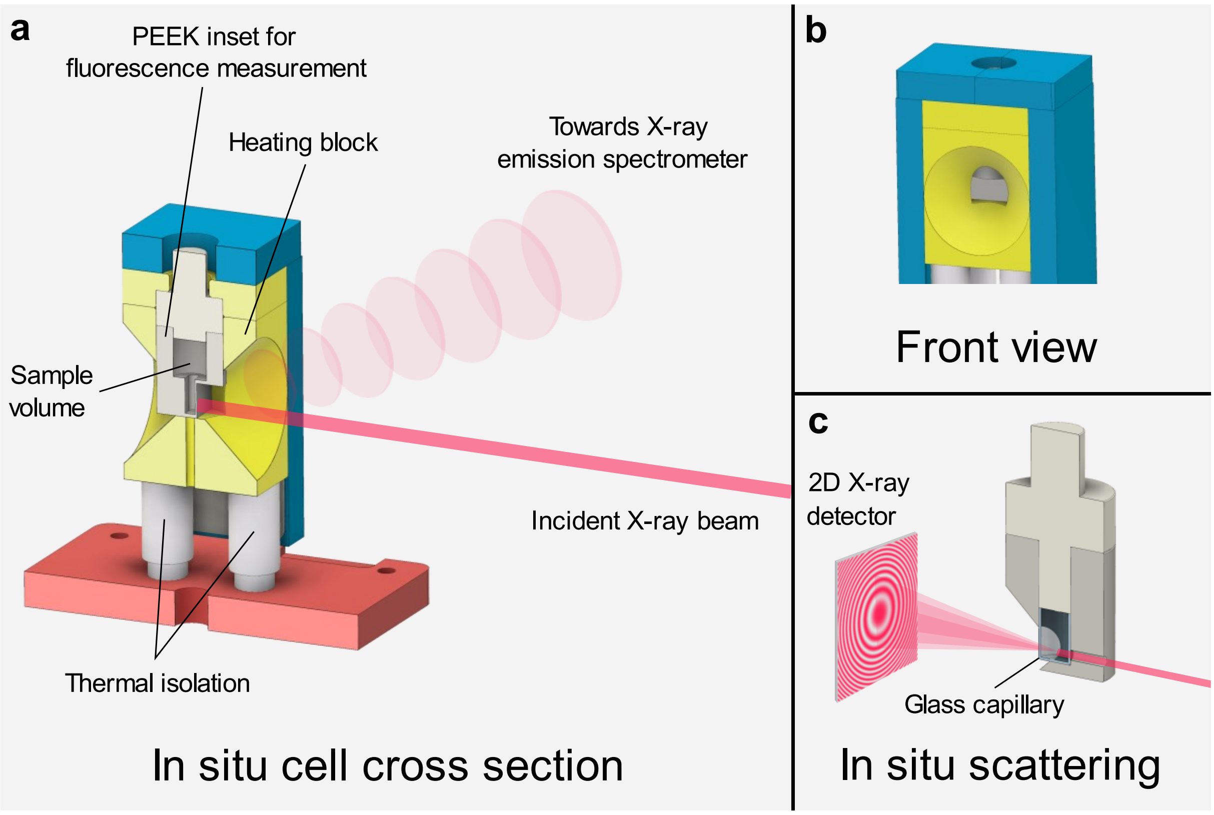

Supplementary Figure 1. Schematics of the reaction cell used for in situ HERFD-XANES and total X-ray scattering studies. a Cross section of the in situ cell with a PEEK inset suitable for fluorescence-detected X-ray absorption spectroscopy. The heating block is made of brass and is heated by PID-controlled heating elements. The PEEK inset is sealed with a PEEK cap that is pressed down by the top brass part. One wall of the PEEK vial has a thickness of 0.2 mm acting as entrance and exit window for the X-rays. Emitted X-rays are detected with a Rowland-circle spectrometer. **b** Front view of the in situ cell with the inset used for fluorescence-detected HERFD-XANES. Additional thermal insulation around the heating block is shown in blue. **c** PEEK inset holding a glass capillary suitable for in situ total X-ray scattering. This inset replaces the one in **a** for total scattering studies. A glass reaction vial is needed here to avoid background diffraction from the semi-crystalline PEEK. A 2D X-ray detector collects the scattered intensities behind the sample. The in situ cell was adopted from reference[1].

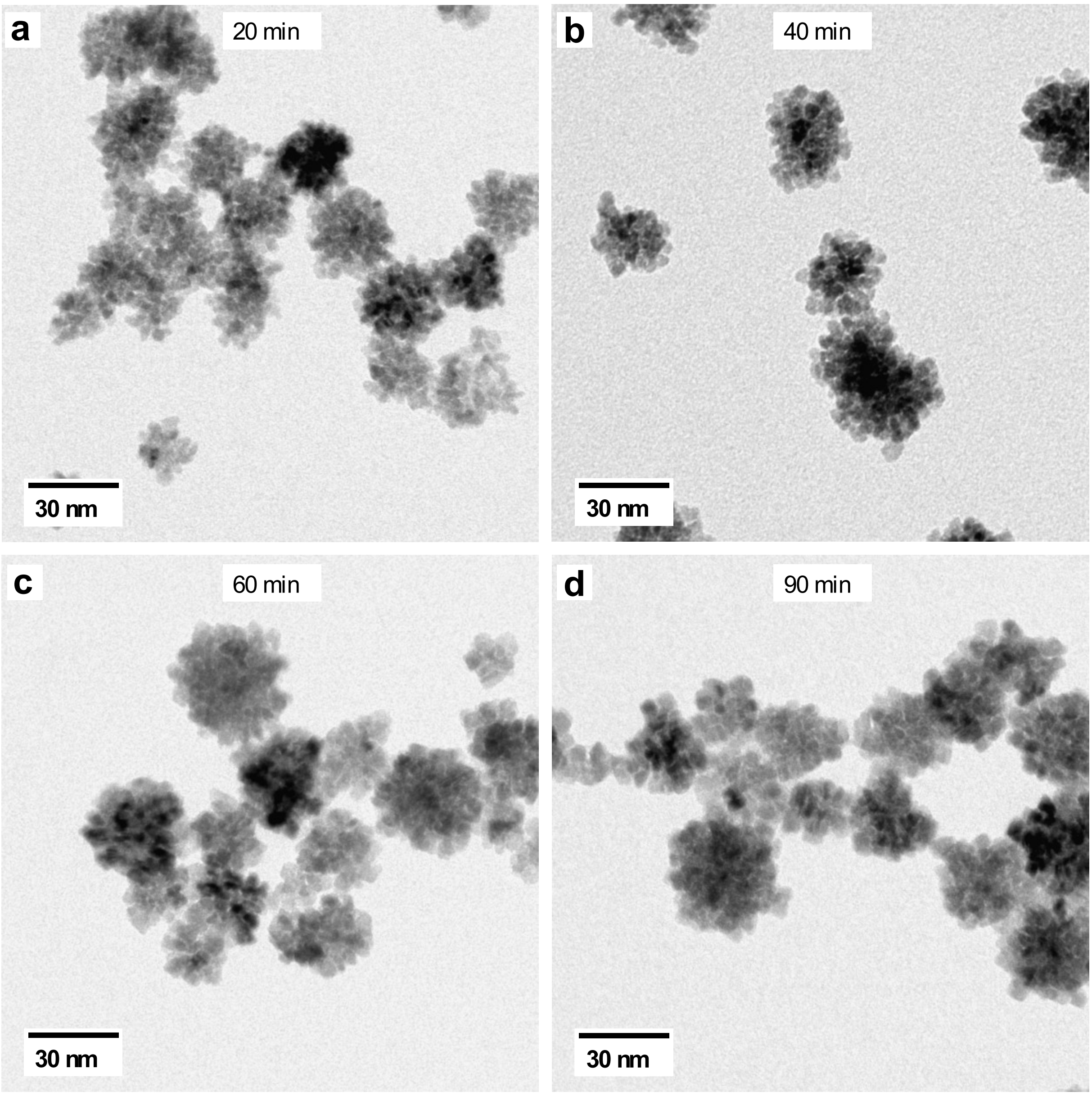

Supplementary Figure 2. TEM images of CoO nanocrystallite assemblies at high magnification. The images were obtained after **a** 20 min, **b** 40 min, **c** 60 min and **d** 90 min reaction time. We used these micrographs for the size analysis of crystallites presented in Supplementary Figure 4.

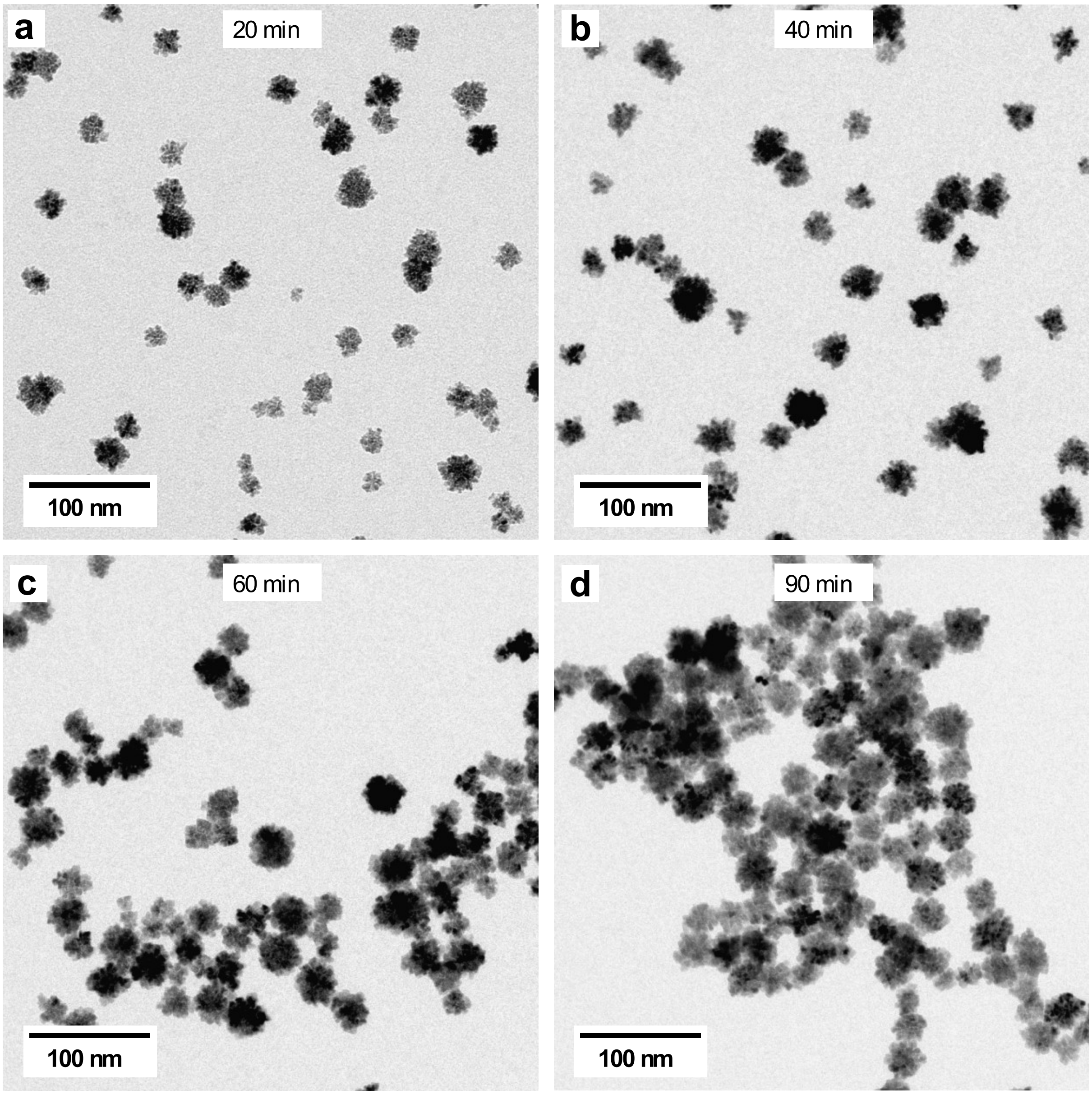

Supplementary Figure 3. TEM images of CoO nanocrystallite assemblies at lower magnification compared to Supplementary Figure 2. The images were obtained after **a** 20 min, **b** 40 min, **c** 60 min and **d** 90 min reaction time. We used these micrographs for the size analysis of assemblies presented in Supplementary Figure 4.

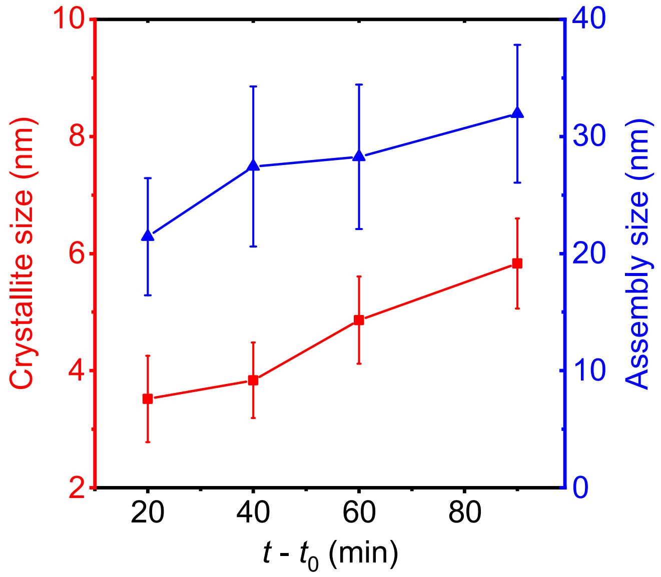

Supplementary Figure 4. Size evolution of polyhedral crystallites and assemblies. The values were obtained from the analysis of time-resolved TEM images in Supplementary Figure 2 and Supplementary **Figure 3**, respectively. At each reaction time, the size analysis is based on 50 crystallites and assemblies. Error bars correspond to the standard deviation of the size distribution.

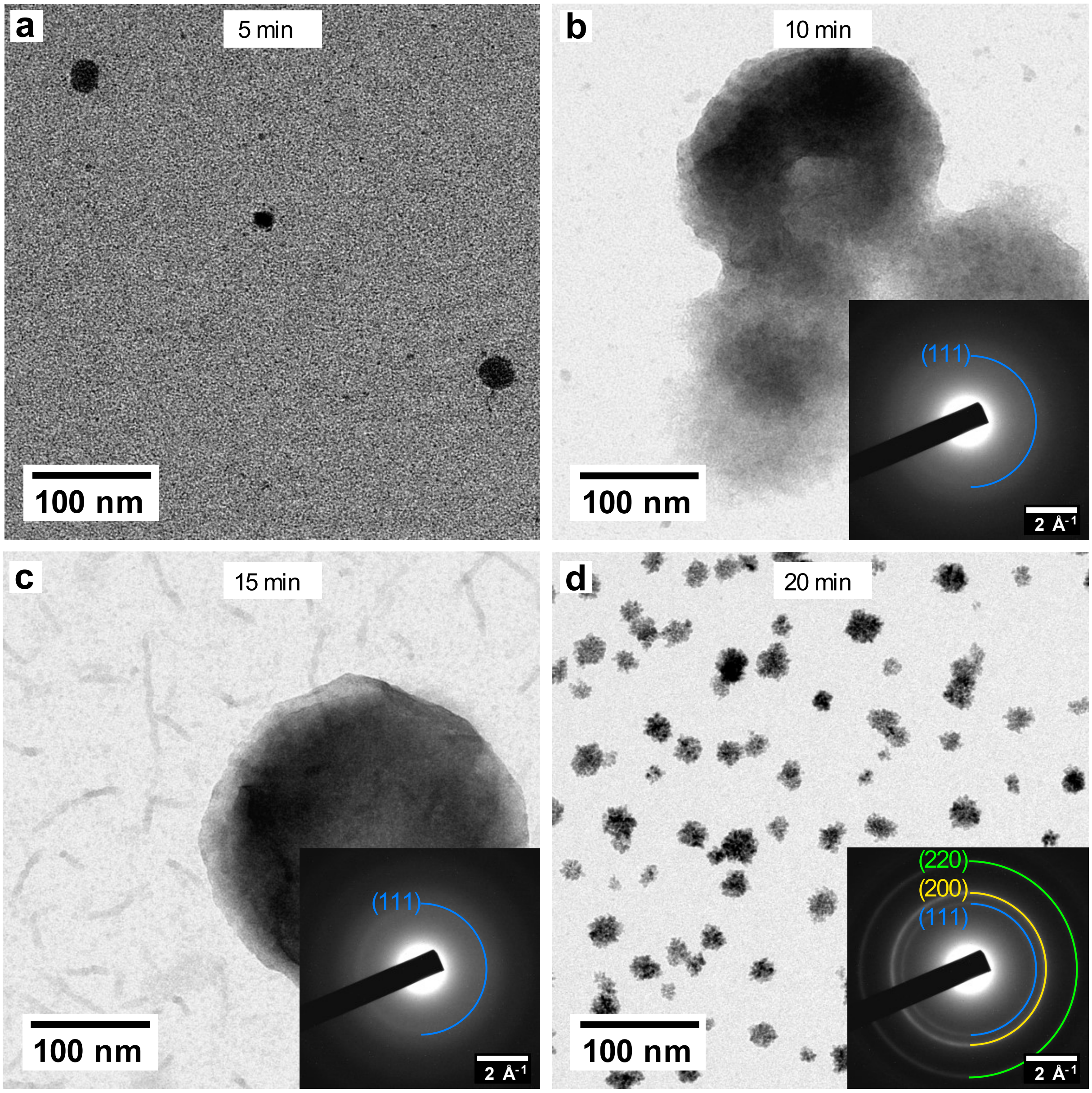

Supplementary Figure 5. TEM images of early reaction stages before (5 to 15 min) and just after (20 min) the onset of crystallization of CoO assemblies (compare Figures 2c and 4d). The images were obtained after **a** 5 min, **b** 10 min, **c** 15 min and **d** 20 min reaction time. Insets show electron diffraction (ED) patterns of different, representative sample regions, respectively, with indexed reflections of the rock salt CoO phase.[2] We account the large structures visible between 10 and 15 min to precipitation of unreacted intermediate $Co(acac)_2$ or precursor $Co(acac)_3$. Only the sample after 20 min shows distinct CoO assemblies, together with a strong crystalline ED signal. In the sample after 15 min, nanosized precipitate is observed. However, the very weak ED signal excludes the presence of final crystalline nanoparticles.

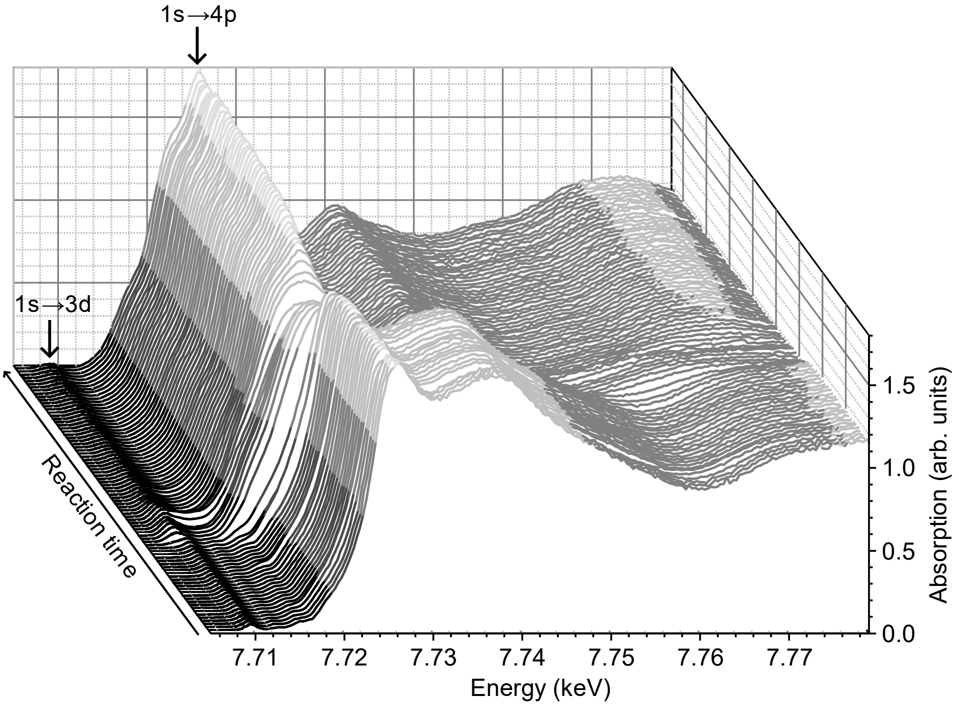

Supplementary Figure 6. Set of Co 1s2p HERFD-XANES spectra measured in situ during the reaction of $Co(acac)_3$ to CoO in BnOH.

Supplementary Notes 1. MCR-ALS method for analysis of in situ HERFD-XANES

The Multivariate Curve Resolution by Alternating Least Squares (MCR-ALS) method[3-5] used for extracting the reaction pathway from the in situ HERFD-XANES data is briefly explained, followed by detailed fit results.

Generally speaking, MCR-ALS is a factor analysis tool, thus it searches for the number and nature of uncorrelated variables explaining the variance in a set of observed, correlated variables. For this, the observed data set needs to be decomposable in the underlying bilinear model given by

$$\mathbf{D} = \mathbf{C}\mathbf{S}^{\mathrm{T}} + \mathbf{E} \tag{1}$$

In Equation (1) and for our case of time-resolved HERFD-XANES spectra, **D** is the experimental data with each row corresponding to a measured spectrum, rows of $\mathbf{S}^{\mathrm{T}}$ are spectra of uncorrelated variables (we call them components) and columns of **C** are the concentration profiles of each component over time. **E** represents variance in the data not explained by the model. After an initial guess for **C** and $\mathbf{S}^{\mathrm{T}}$, which we do by means of the purest variables detection method,[4, 6] a set of linear equations is iteratively solved, alternatingly keeping **C** or $\mathbf{S}^{\mathrm{T}}$ constant until the change in the standard deviation of **E** falls below a certain convergence criterion. Additional constraints can be imposed on the optimization problem (spectral and/or concentration dimension) to facilitate convergence, one being non-negativity and the other one being unimodality.

Quality estimates of a converged fit arise from the unexplained residuals **E**, or the difference between the experimental data and the bilinear model, with the lack of fit given by

$$\text{lack of fit (\%)} = 100\sqrt{\frac{\sum_{i,j} e_{ij}^2}{\sum_{i,j} d_{ij}^2}} \tag{2}$$

where d_{ij} is a data matrix element and e_{ij} is the corresponding element of the residuals matrix **E**. Additionally, the variance explained in the model can be estimated from

$$R^2 = \frac{\sum_{i,j} d_{ij}^2 - \sum_{i,j} e_{ij}^2}{\sum_{i,j} d_{ij}^2} \tag{3}$$

and the standard deviation of the residuals is given by

$$\sigma = \sqrt{\frac{\sum_{i,j} e_{ij}^2}{n\,m}} \tag{4}$$

where n, m refer to the dimensions of **D**. For the MCR-ALS analysis in the main article, we show the quality estimates in Supplementary Table 1.

Supplementary Table 1: MCR-ALS fit quality estimates.

**fit quality estimate**	**value**
lack of fit (%)	2.088
R^2	99.956
σ	0.017

The MCR-ALS optimization can only start after the dimensions of **C** and $\mathbf{S}^{\mathrm{T}}$ have been defined, thus the number of components must be defined beforehand. For this purpose we use Singular Value Decomposition (SVD) which computes a set of eigenvalues and corresponding eigenvectors from the

input data set.[3] Both of them contain information to determine the number of components. A higher eigenvalue means that the respective component explains more variance of the data, while the graphical representation of the eigenvector of a meaningful component should display a distinctive profile, independent of its absolute values. Such meaningful components correspond to HERFD-XANES spectra of chemical compounds in the reaction mixture.

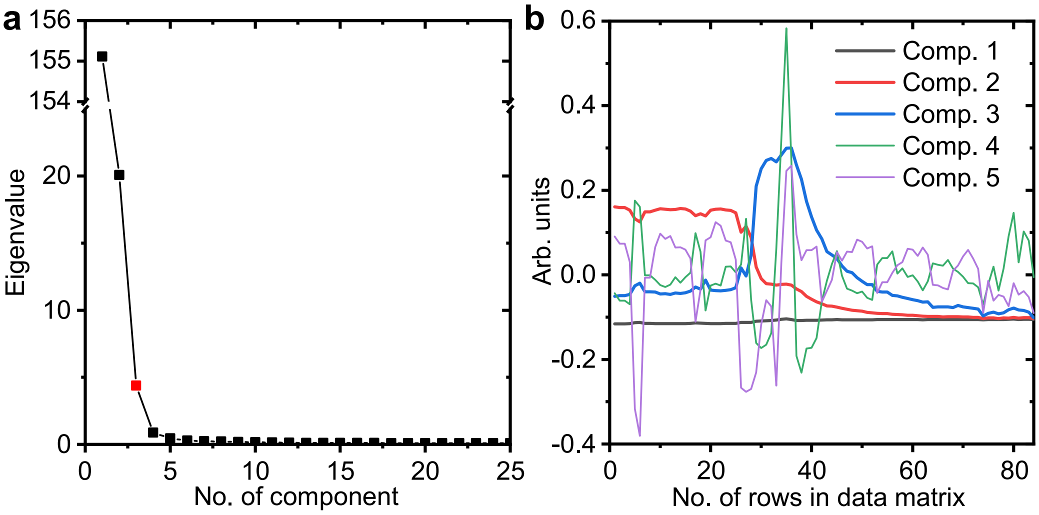

Supplementary Figure 7. SVD results of the in situ HERFD-XANES data set. a Scree plot of eigenvalues with elbow starting at component 3. Small, linearly aligned eigenvalues correspond to noise. **b** Eigenvector representation with components 1 to 3 (bold lines) showing distinctive profiles.

We show the results of SVD of the in situ HERFD-XANES data set in Supplementary Figure 7. The scree plot of the eigenvalues in Supplementary Figure 7a has an elbow starting after component 3. Low eigenvalues after the elbow starting with component 5, which align linearly, correspond to the noise in the data. The exact eigenvalues are given in Supplementary Table 2. Additionally, looking at the eigenvector representations in Supplementary Figure 7b, we see clear profiles only for components 1 to 3, while components 4 and 5 only punctually exceed the noise level, which we assign to fluctuations due to changing positions of the incident beam on the reaction container throughout the collection of the in situ data set. The wall thickness of the reaction container could vary in a certain range, and we have to note that no stirring was applied during the in situ studies. From both measures combined, we conclude that there are 3 independent components in the data set. Additionally, MCR-ALS optimization with 4 and more components would not give physically meaningful results.

Supplementary Table 2: The first ten eigenvalues calculated by SVD of the in situ HERFD-XANES data matrix.

**no. of component**	**eigenvalue**
1	155.112
2	20.085
3	4.377
4	0.884
5	0.45
6	0.295
7	0.226
8	0.206
9	0.179
10	0.153

Supplementary Notes 2. HERFD-XANES self-absorption correction

Self-absorption can affect the intensities of features in fluorescence-detected XAS. In order to correct for this, we apply the method described by Booth and Bridges[7] expressed by Equation (5):

$$\frac{I_\mathrm{f}}{I_0} \equiv I_\mathrm{f}^0 = \frac{\varepsilon_\mathrm{a}\mu_\mathrm{a}}{\mu + g\mu_\mathrm{f}}\left[1 - e^{-\left(\frac{\mu}{\sin\varphi}+\frac{\mu_\mathrm{f}}{\sin\theta}\right)d}\right] \tag{5}$$

Here, I_0 and I_f are the incident and fluorescence intensities, $\varepsilon_\mathrm{a}(E)$ is the fluorescence efficiency per unit solid angle, $\mu_\mathrm{a}(E)$ is the absorption coefficient due to core hole excitation, $\mu(E)$ is the total absorption coefficient, $\mu_\mathrm{f} = \mu(E_\mathrm{f})$ is the absorption coefficient at the detected fluorescence energy, φ and θ are the angles of incident and outgoing X-rays with respect to the sample surface, $g \equiv \sin\varphi\,/\,\sin\theta$, and d is the thickness of the sample. Energy dependencies are implicit in all equations. In our case of HERFD-XANES, $\varphi = \theta = 45°$, $\varepsilon_\mathrm{a} \approx \mathrm{const.}$ and if we assume a thick sample with respect to the absorption length, we can say that

$$\left[1 - e^{-\left(\frac{\mu}{\sin\varphi}+\frac{\mu_\mathrm{f}}{\sin\theta}\right)d}\right] \approx 1$$

and we can additionally assume that $\mu_\mathrm{a}(E) \approx \mu(E)$. Thus, Equation (5) simplifies to

$$I_\mathrm{f}^0 \approx \frac{\varepsilon_\mathrm{a}\mu}{\mu + \mu_\mathrm{f}}$$

which we can rearrange to

$$\mu \approx \frac{\mu_\mathrm{f} I_\mathrm{f}^0}{\varepsilon_\mathrm{a} - I_\mathrm{f}^0} \tag{6}$$

For the self-absorption corrected fluorescence intensity, $I_{\mathrm{f}_\mathrm{corr}}^0$, we can say that $I_{\mathrm{f}_\mathrm{corr}}^0 \sim \mu$ for $I_{\mathrm{f}_\mathrm{corr}}^0 \ll 1$ and since we are not interested in absolute values for the absorption coefficient, we can use Equation (6) to perform self-absorption correction without knowledge of μ_f or ε_a.

We do self-absorption correction based on Equation (6) for the CoO powder reference in a way that we minimize the difference to the MCR-ALS spectrum of CoO. Due to the relatively low concentration of nanoparticles in the solution, we can assume the MCR-ALS spectrum not to contain any significant self-absorption. The corrected reference spectrum, after re-normalizing the edge step, is shown in Supplementary Figure 8 together with the uncorrected MCR-ALS spectrum.

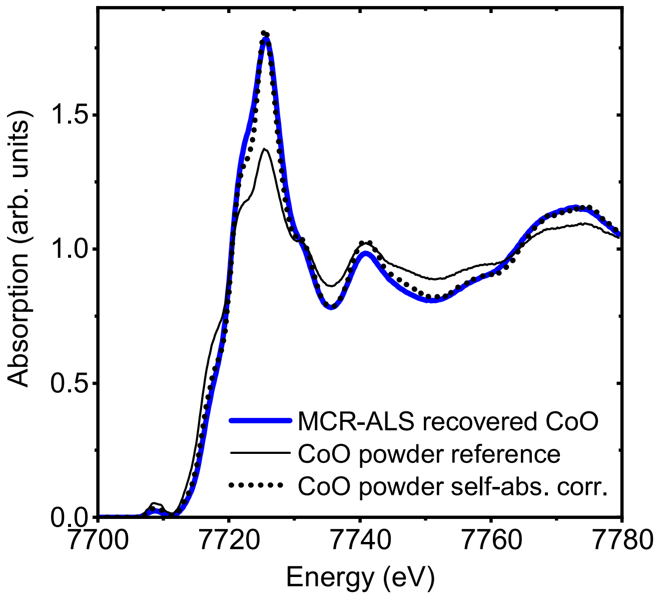

Supplementary Figure 8. Co 1s2p HERFD-XANES spectrum of the CoO powder reference before and after self-absorption correction. The spectrum of CoO recovered by MCR-ALS is also shown.

Supplementary Notes 3. Kinetic fit of MCR-ALS results

The reaction of $Co(acac)_3$ with BnOH at 160 °C includes two steps, a reduction to $Co(acac)_2$ followed by the formation of CoO. The concentration profiles obtained from the MCR-ALS analysis give a hint on the kinetics of the reaction steps. Supplementary Figure 9 shows plots of the concentrations of the precursor and intermediate in natural logarithmic and reciprocal scale. Arrows in Supplementary Figure 9a mark changes in the beam position on the reaction container by a relatively long distance during the collection of the data set. The approximately linear decay in natural logarithmic scale within the intervals between the position changes is a visual indication of pseudo first order kinetics. A previous study on the formation on ZnO nanoparticles from zinc acetate in BnOH reported similar kinetics.[8, 9]

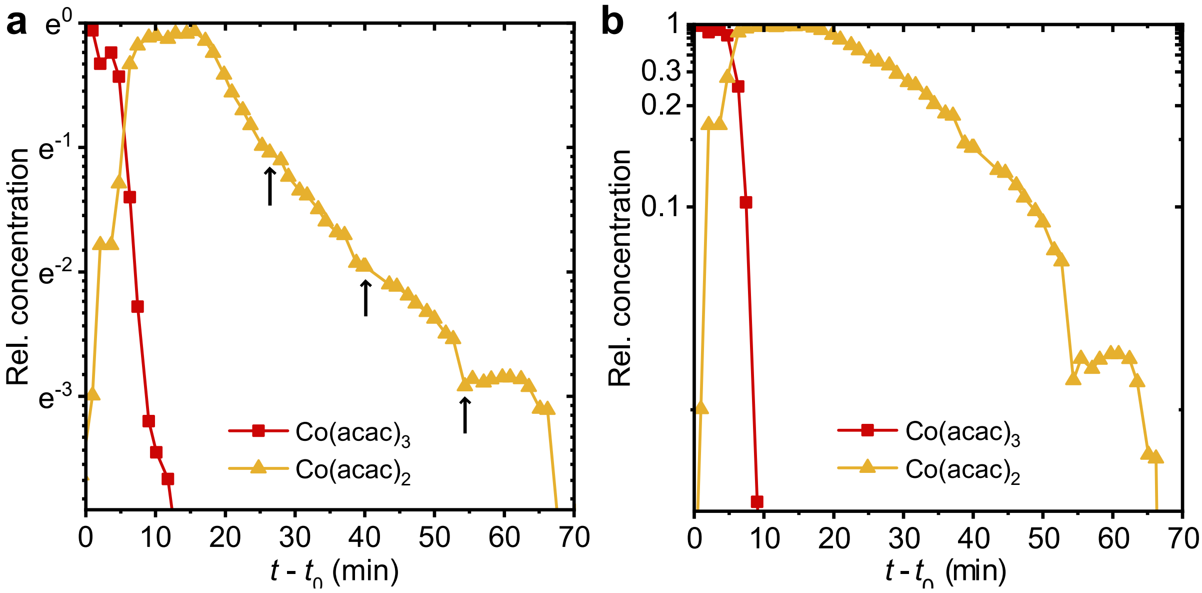

Supplementary Figure 9. Relative concentrations of the precursor and the intermediate. The same values are plotted **a** in natural logarithmic scale and **b** reciprocal scale. Arrows in **a** mark large changes in the position of the X-ray beam on the reaction container. The linear decay in the intervals between the position changes indicates pseudo first order kinetics.

Supplementary Notes 4. Calculation of XANES spectra using the FEFF code

In addition to reference measurements, we verify the reaction components identified by MCR-ALS with theoretical calculations of Co K-edge XANES spectra that we obtain using the FEFF code.[10] From Supplementary Figure 10, we see that the calculated spectra of the reaction precursor, $Co(acac)_3$, and the product, CoO, are in good agreement with the experimentally obtained spectra of these compounds. Additionally, we see that the 3d density of states (DOS) of the cobalt ion mainly determines the shape of the pre-edge transitions. Thus, the pre-edge features arise from hybridization of Co 4p and 3d states, allowing to a certain extent 1s→3d quadrupole transitions. The calculated spectrum of CoO is missing a feature ~8 eV above the absorption edge compared to the experiment, which we account to a final state effect in the HERFD-XANES measurement not reproducible with FEFF. Standard transmission XANES at the Co K-edge also excludes this feature.[11]

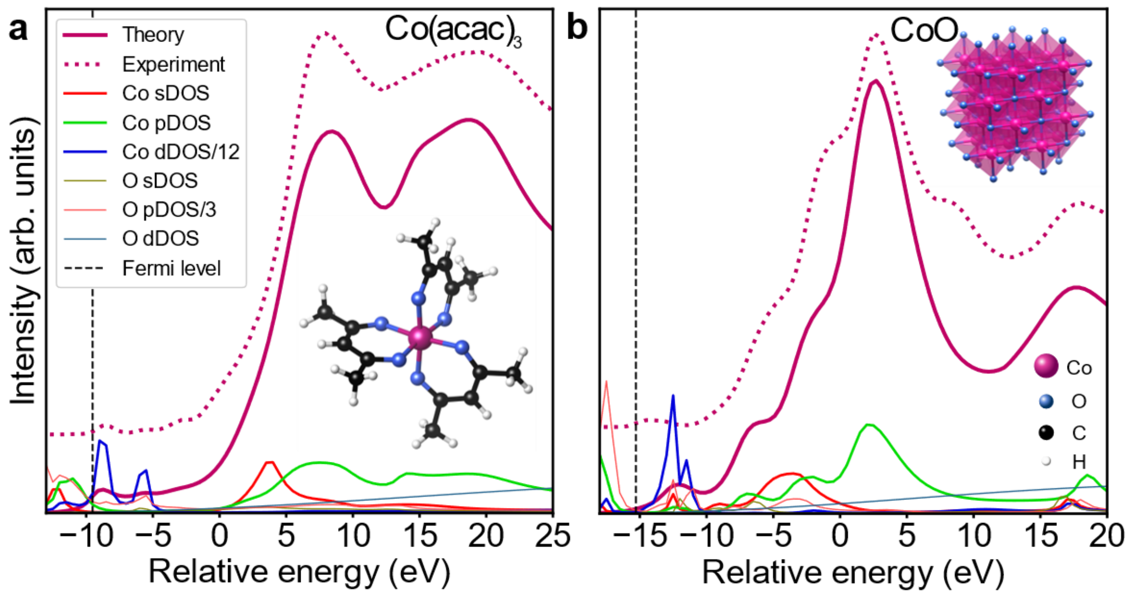

Supplementary Figure 10. Theoretical Co K-edge XANES spectra calculated using the FEFF code.[10] **a** Theoretical spectrum of $Co(acac)_3$. **b** Theoretical spectrum of CoO. The spectra are plotted together with the local DOS for s, p and d states. The experimental HERFD-XANES spectrum of the respective compound is shown as dotted line. The energy scale is relative to the absorption edge E_0. The crystal structure of CoO and the atomic positions in $Co(acac)_3$ were taken from references[2, 12], respectively.

Furthermore, FEFF calculations allow deeper insight into the structure of the reaction intermediate, $Co(acac)_2$. Previous theoretical and experimental studies revealed a number of conformations of the $Co(acac)_2$ molecule depending on its aggregate state and environment. Diffraction experiments showed that in the crystalline state, the molecule adopts the tetrameric form $Co_4(acac)_8$[13, 14]. DFT calculations of the isolated molecule indicate a small energy difference between the square-planar and tetrahedral conformation, thus the interaction with the solvent plays an important role for the actual structure.[15] In Supplementary Figure 11, we present FEFF calculations for the possible structures of $Co(acac)_2$. It is clear that only in the case of an octahedrally coordinated Co^{2+} ion, the theory is in good agreement with the experiment. We conclude that for the reference that was measured as a powder, the molecule forms tetramers with octahedral coordination (c.f. Supplementary Figure 11d). In solution, the Co^{2+} ion is additionally coordinated by oxygen atoms of two solvent molecules, forming a bis-adduct with octahedral coordination[13] (c.f. Supplementary Figure 11c). Note that since the actual coordination geometry of $Co(acac)_2$ in BnOH is unknown, we used water as a model coordinating solvent for the FEFF calculations.

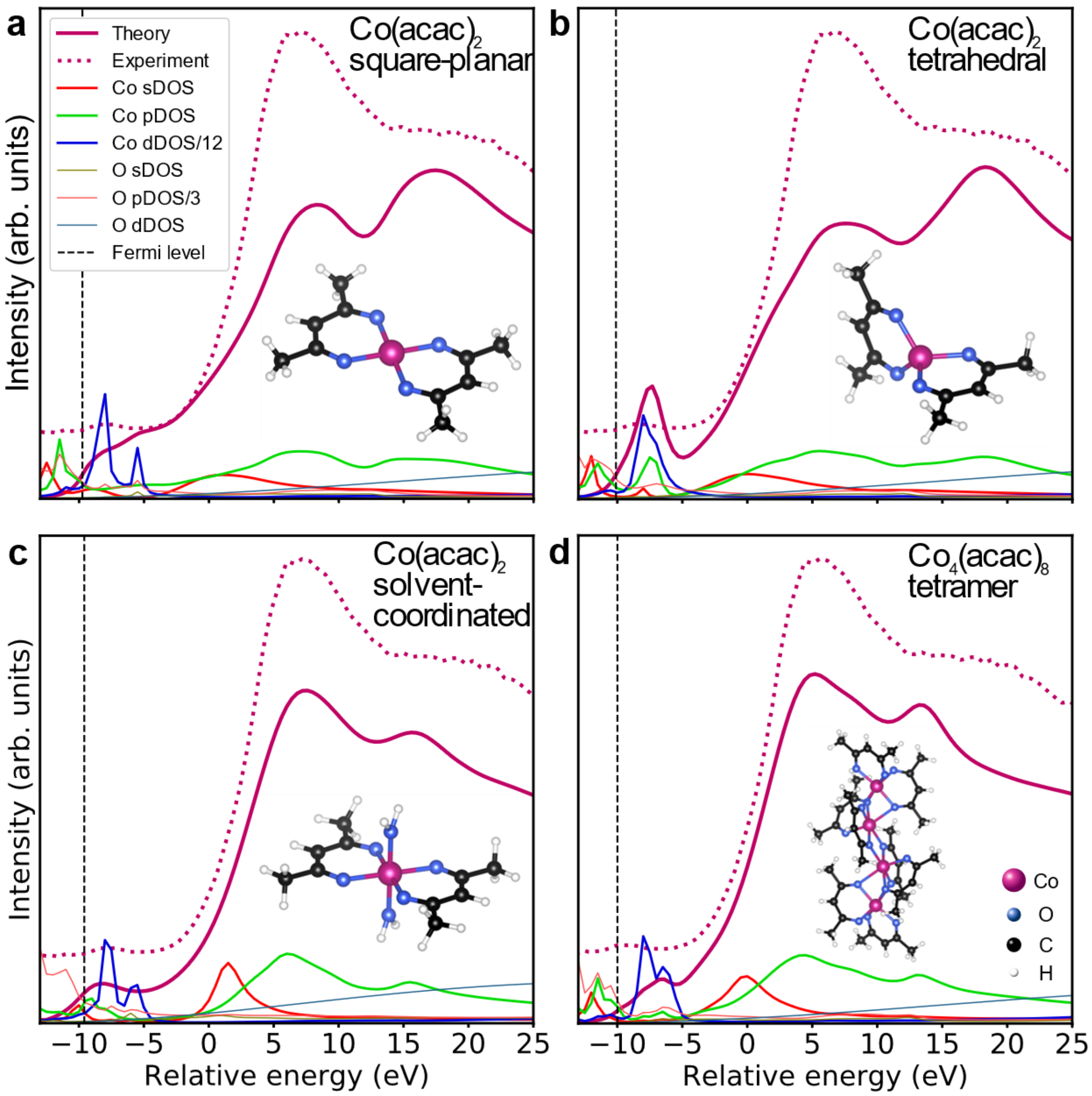

Supplementary Figure 11. Theoretical XANES spectra of Co(acac)$_2$ calculated using the FEFF code.[10] We compare **a** square-planar, **b** tetrahedral, **c** solvent-coordinated with water as a model solvent and **d** tetrameric geometry. The spectra are plotted together with the local DOS for s, p and d states. The experimental HERFD-XANES spectrum of Co(acac)$_2$ powder is shown as dotted line. The energy scale is relative to the absorption edge E_0. Vertical dashed lines indicate the position of the Fermi level. The atomic positions in square-planar and tetrahedral Co(acac)$_2$ were taken from reference[15] and the structure of the tetramer was adopted form reference[14].

We used the FEFF code[10] in version 9.6.4 with the Hedin-Lundquist energy dependent exchange correlation potential for all calculations. The settings are listed in Supplementary Table 3. Note that no instrumental broadening (EXCHANGE card setting v_i) needed to be applied since the experimental spectra were measured in HERFD mode. For the Co$_4$(acac)$_8$ tetramer, we averaged calculations with each of the Co ions set as the absorber, since their environments in the tetramer are not equivalent.

Supplementary Table 3: Settings for the FEFF calculations shown in Supplementary Figure 10 and Supplementary Figure 11.

**FEFF card**	**ATOMS**	**COREHOLE**	**EXCHANGE**		**SCF**		**FMS**	
**compound**	**number**	**type**	v_r	v_i	r_{scf}	l_{scf}	r_{fms}	l_{fms}
$Co(acac)_3$	43	none	3.5	0	6.0	1	6.0	1
$Co(acac)_2$ tetrahedral	29	none	3.5	0	6.0	1	6.0	1
$Co(acac)_2$ sq.-planar	29	none	3.5	0	6.0	1	6.0	1
$Co(acac)_2$ solv. coord.	35	none	3.5	0	6.0	1	6.0	1
$Co(acac)_2$ tetramer	116	none	3.5	0	6.0	1	6.0	1
CoO	147	none	3.5	0	6.3	0	6.3	0

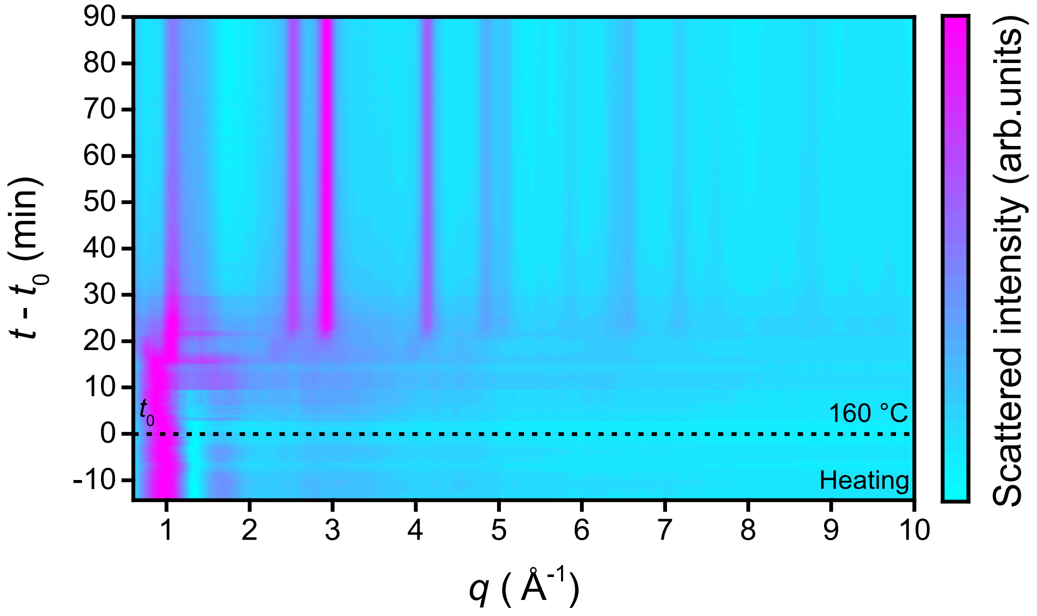

Supplementary Figure 12. In situ time-resolved background-subtracted total X-ray scattering measurements for the reaction at 160 °C. The heating step involves heating from room temperature to 60 °C, 5 min at 60 °C, and the heating from 60 to 160 °C. The normalized intensity of the total X-ray scattering data for the final product at 90 min and the background are shown in Supplementary Figure **13**.

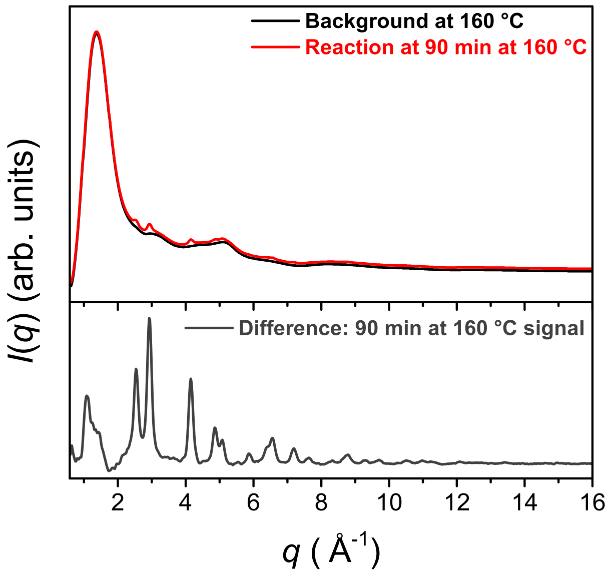

Supplementary Figure 13. Normalized total X-ray scattering data for the reaction at 90 min after 160 °C and the background (glass vial filled with BnOH) at 160 °C. The signal to background ratio is very low, mainly due to the low concentration of the precursor. The difference curve reveals the signal of the reaction product after 90 min showing CoO features.

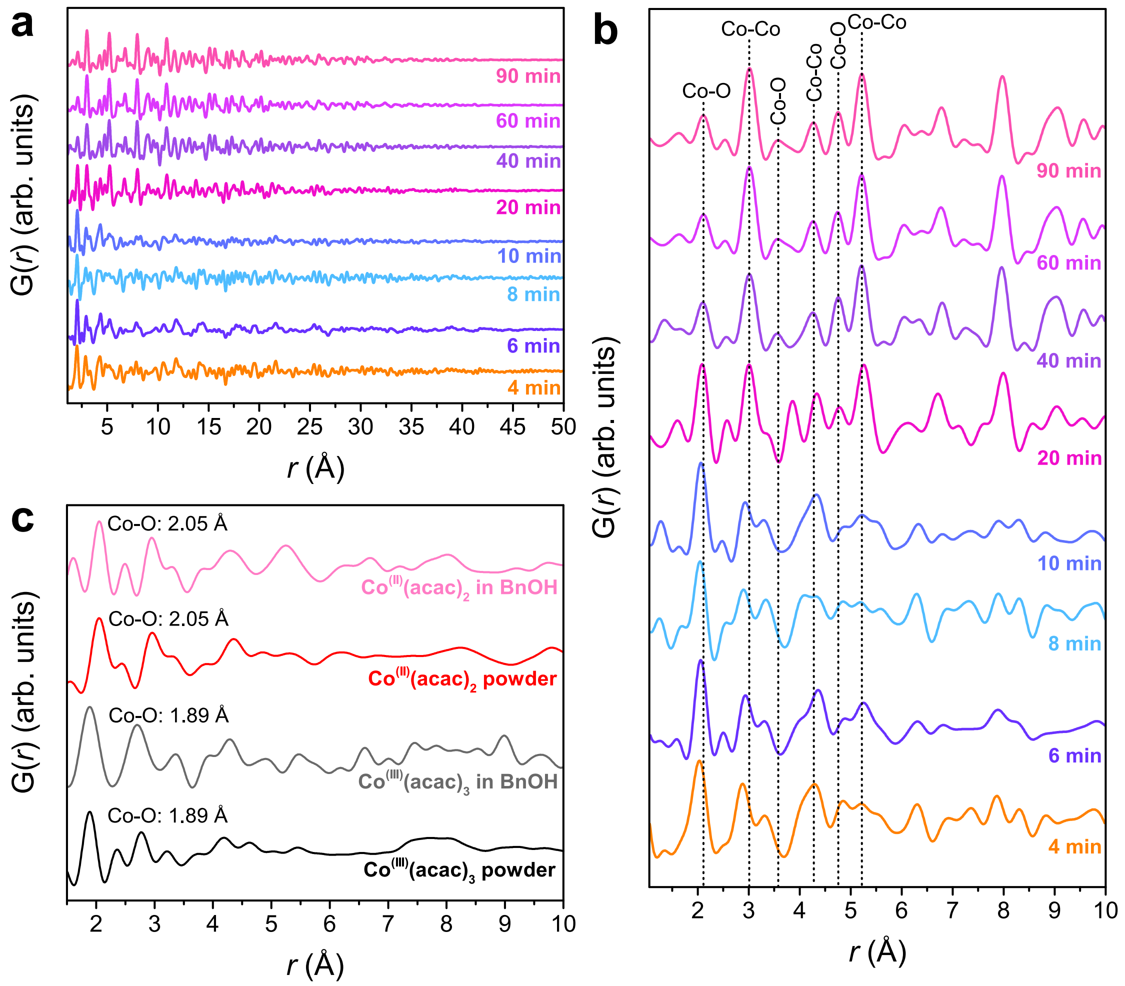

Supplementary Figure 14. Ex situ PDFs for the synthesis at 160 °C. **a** PDFs at different reaction times. **b** Zoom of **a** showing the PDFs at the local ordering region. The dashed lines show the first coordination shells of CoO. **c** PDFs of the $Co(acac)_3$ and $Co(acac)_2$ references. The measurements were performed for the powders and 0.1 M solutions in BnOH.

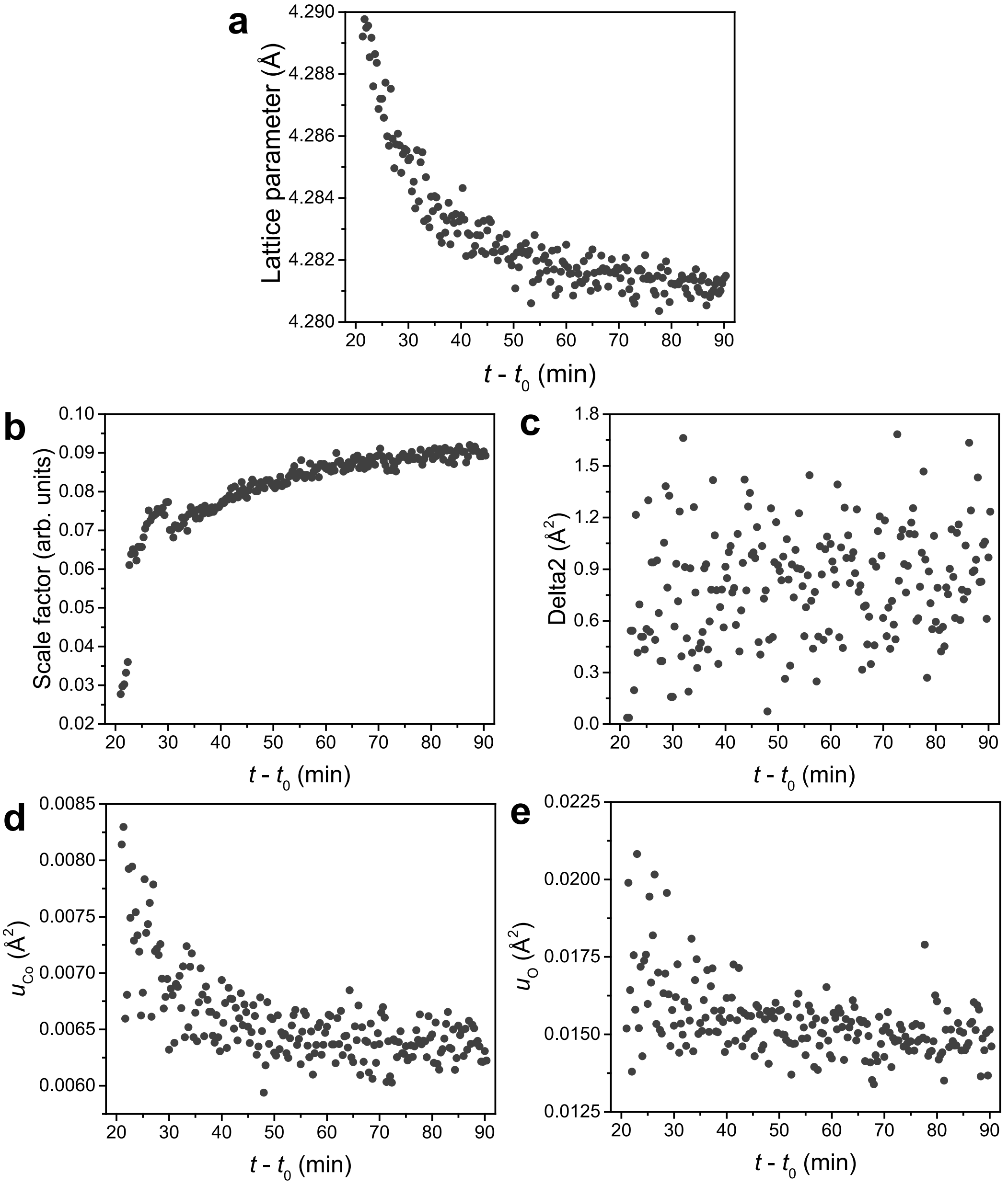

Supplementary Figure 15. PDF refinement results for the in situ reaction at 160 °C. Evolution of **a** lattice parameter *a*, **b** scale factor, **c** the quadratic atomic correlation factor δ_2, **d** isotropic atomic displacement parameter u_{Co} for cobalt, and **e** isotropic atomic displacement parameter u_O for oxygen, as a function of the reaction time. The data were obtained from the sequential refinement of time-resolved PDFs. The lattice parameter shown in **a** rapidly decreases as the reaction progresses and fluctuates around 4.28 Å throughout most of the reaction time. However, since the co-existence of $Co(acac)_2$ and CoO induces a misfit in the low *r* region before 40 min (Supplementary Figure 17a), this strong decrease may not be a real trend.

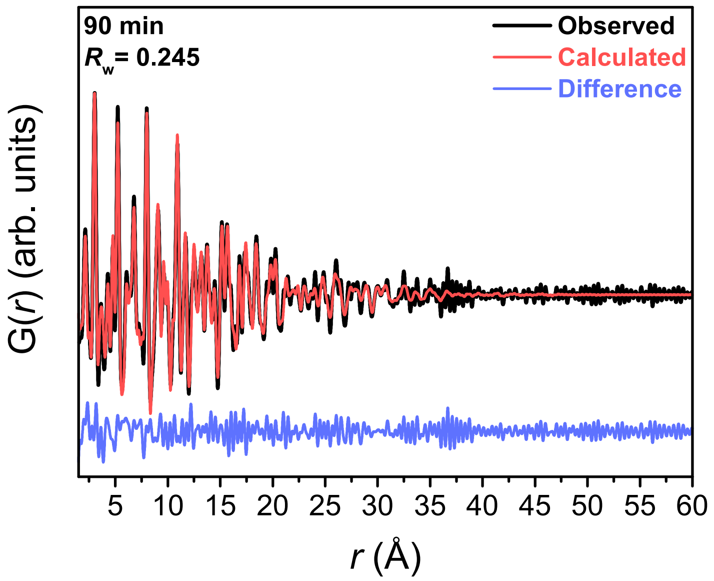

Supplementary Figure 16. PDF refinement with higher *r* range. The plot shows a fit between experimental and calculated G(*r*) for the PDF obtained at 90 min at 160 °C using a higher *r* range of 1.5 to 60 Å compared to Figure 5c. No correlations are seen beyond 40 Å but noise. Consequently, the R_w value increases compared to the fit up to 40 Å, but no significant changes are observed for the other refined parameters, indicating that the fit up to 40 Å is suitable. The refined value for *a* is 4.281 Å, for the scale factor is 0.0891, for δ_2 is 1.25 Å^2, for u_{Co} is 0.0062 Å^2, for u_O is 0.0146 Å^2, and for the sp-diameter is 64 Å.

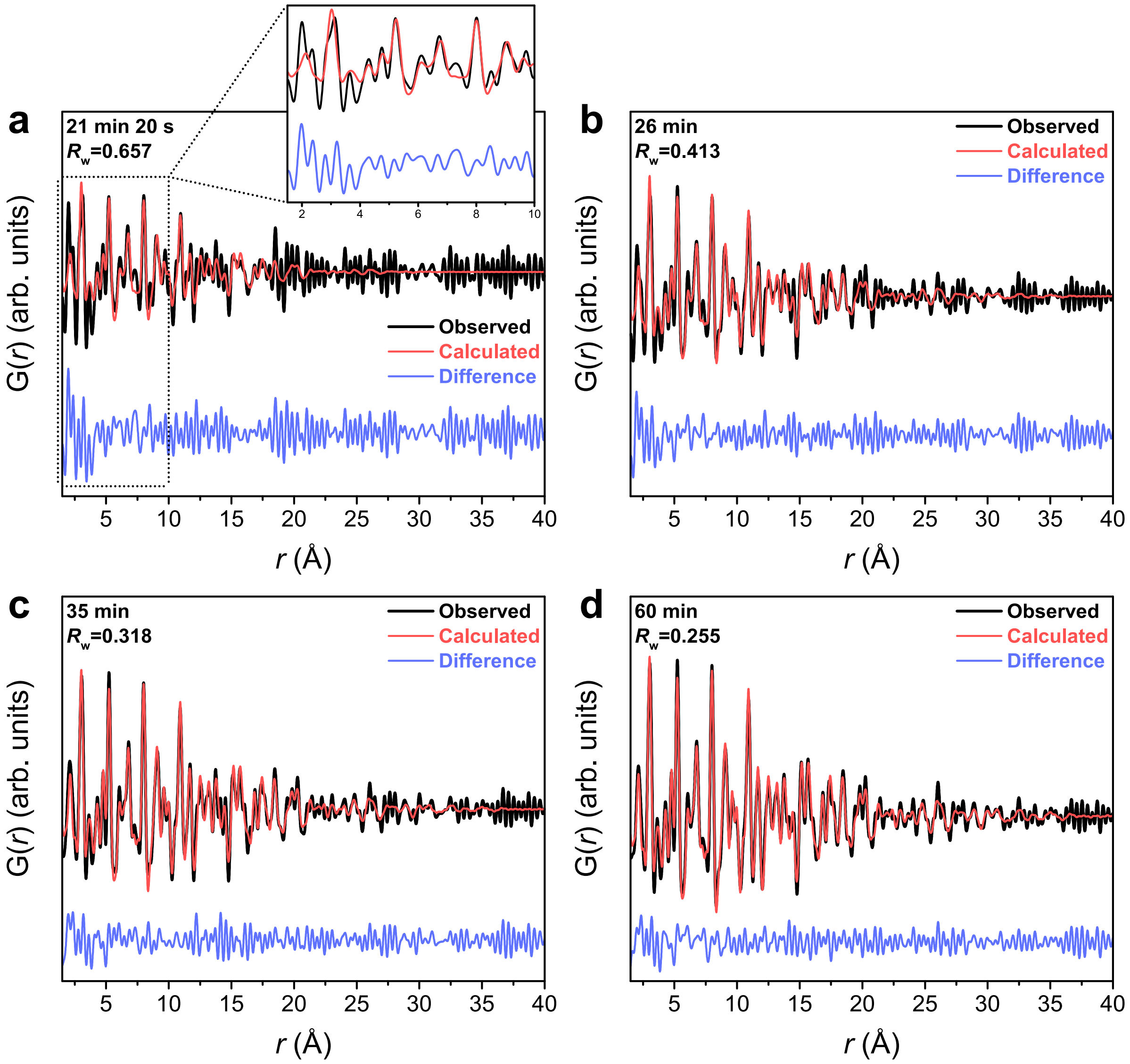

Supplementary Figure 17. Fit between experimental and calculated G(*r*) using the cubic CoO phase. **a** Fit for the in situ reaction at 160 °C at 21 min 20 s reaction time, the inset shows the short range order fit, demonstrating the lack of fit for the first peak probably due to the presence of $Co(acac)_2$. **b** Fit for 26 min, **c** 35 min, and **d** 60 min reaction time.

Supplementary Notes 5. In situ total scattering studies of the reaction at 140°C

The CoO reflections in time-resolved in situ total scattering data at 140 °C (Supplementary Figure 18a) occur after 64 min reaction time, which is 44 min later than at 160°C. Similarly, the long-range order correlations in the time-resolved PDFs emerge later. (Supplementary Figure 18b,c). We evaluate the structural parameters by the same sequential refinement method starting from the final product after 210 min and tracing the signal back towards earlier reaction times. The refinement results are depicted in Supplementary Figure 19. The evolution of the sp-diameter over time provides a value of ~26 Å at 70 min, which progressively increases and reaches ~64 Å at 210 min. Therefore, although the reaction at 140 °C yields CoO nanoparticles with a similar particle size as the reaction at 160 °C, the reaction takes ~2.3 times longer.

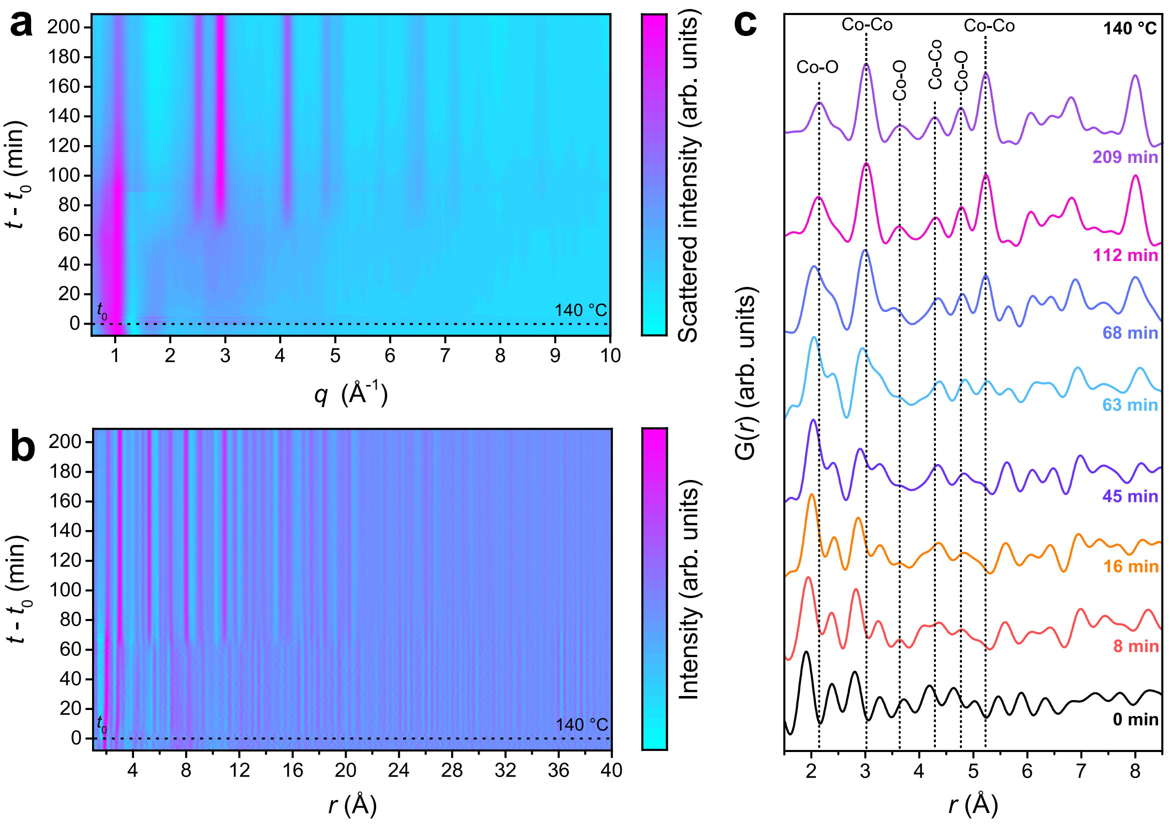

Supplementary Figure 18. In situ total X-ray scattering data for the reaction at 140 °C. a In situ time-resolved background-subtracted $I(q)$ data. **b** In situ time-resolved PDFs. The heating steps in **a** and **b** involve the heating from room temperature to 60 °C, 5 min at 60 °C, and the heating from 60 to 140 °C. **c** PDFs at the local ordering region for selected reaction times of the in situ data.

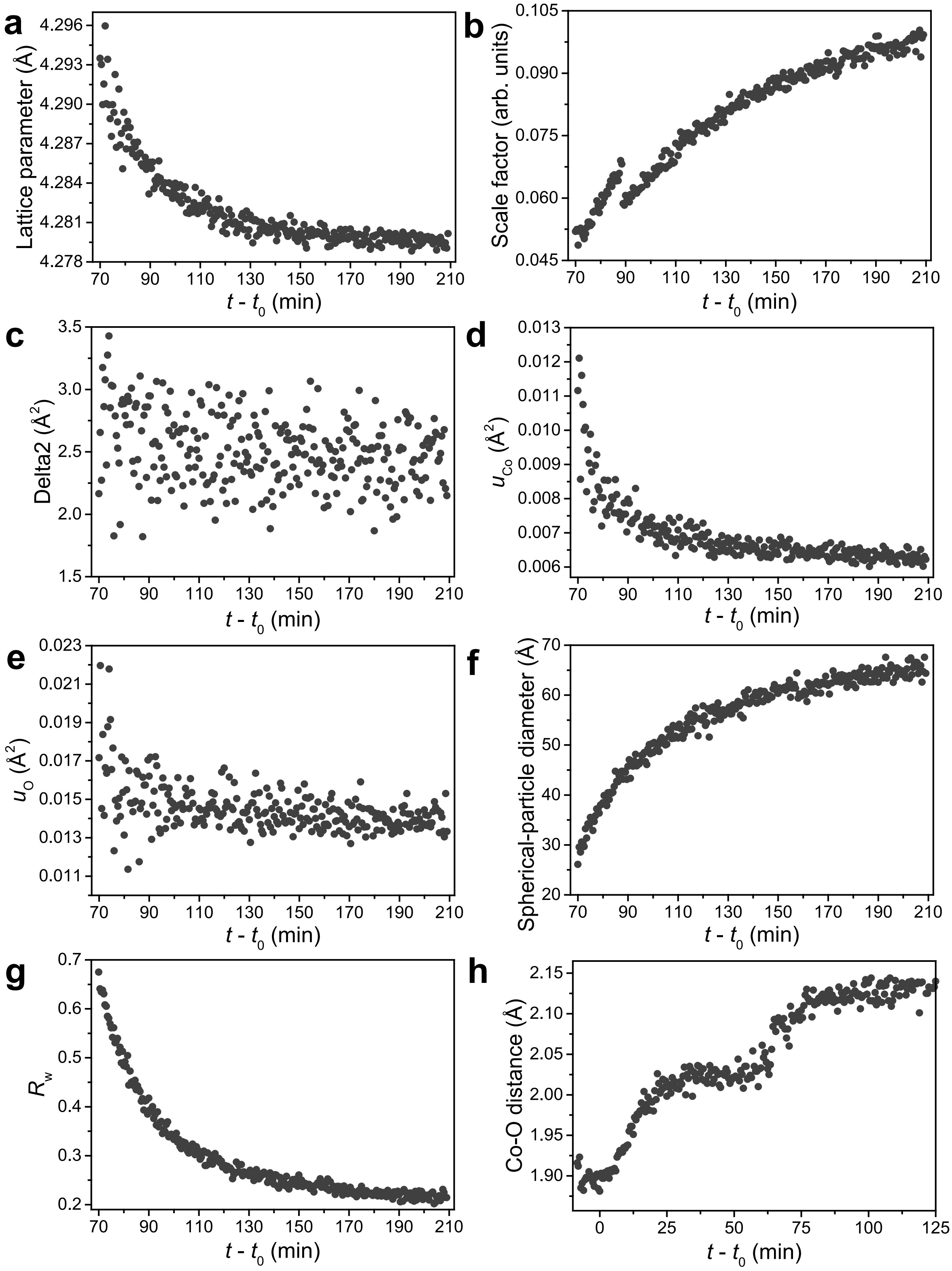

Supplementary Figure 19. Additional refined PDF parameters. a-**g** Refinement results for the in situ reaction at 140 °C. Evolution of **a** lattice parameter *a*, **b** scale factor, **c** the quadratic atomic correlation factor δ_2, **d** isotropic atomic displacement parameter u_{Co} for cobalt, **e** isotropic atomic displacement parameter u_{O} for oxygen, **f** sp-diameter, and **g** R_w, as a function of the reaction time. The data were obtained from the sequential refinement of time-resolved PDFs. **h** Changes in the shortest Co-O bond over the reaction course at 140 °C.

Supplementary Notes 6. SAXS data analysis

Homogeneous spherical model:

The low q regime of the SAXS data was analyzed with a model form factor intensity describing homogeneous spherical particles. The form factor is related to the particle diameter $D = 2R$ by

$$\Psi(q,R) = \frac{3(\sin(qR) - qR\cos(qR))}{(qR)^3}$$

The scattering intensity of a particle in a solvent with scattering contrast $\rho_{\text{particle}} - \rho_{\text{solvent}}$ is

$$I(q) = \text{const} \cdot c \cdot V \cdot \left[3(\rho_{\text{particle}} - \rho_{\text{solvent}}) \cdot \frac{\sin(qR) - qR\cos(qR)}{(qR)^3}\right]^2 + \text{background}$$

$V = \frac{4\pi}{3}R^3$ is the volume of the particle and c is the volume fraction of the particles, which can be determined in absolute units if the instrumental scaling constant (const) and the scattering contrast are determined e.g. by calibration measurements of standard substances.

The model is included in the SasView software package, version 4.2.2. We used the population-based DREAM algorithm with 10^4 samples for fitting. Radii were drawn from a Schulz-Zimm polydispersity distribution.[16] The fitted data range is shown as a solid curve in Figure 6. Resulting particle diameter values are given in Supplementary Table 4.

Supplementary Table 4: Particle diameters *D* and polydispersities Δ*D*/*D* obtained from the model fit.

reaction time	20 min	40 min	60 min	90 min
D	21 nm	45 nm	52 nm	58 nm
**Δ*D*/*D***	0.25	0.19	0.18	0.19

To quantify the concentration of assemblies obtained at the beginning and end of assembly formation, we recorded SAXS data of aliquots extracted at 20 and 90 min reaction time with our laboratory SAXS setup at LMU. Here, we obtain the scattering intensity on absolute scale (cm^{-1}), by using a high dynamic range, photon counting detector, and normalizing the intensity as

$$I(q) = \frac{I_{\text{raw}}(q)}{I_1 \Omega t_{\text{m}} t}$$

$I_{\text{raw}}(q)$ is the integrated SAXS intensity in units of photons. I_1 is the transmitted direct beam intensity through the sample in photons/second. Ω is the solid angle of a detector pixel. t_{m} is the measurement time in seconds and t the sample thickness in cm. This procedure obviates the need for measuring an intensity standard substance and the instrumental scaling constant (const) equals 1.

The data and a least-squares fit with a model of homogeneous spherical particles are shown in Supplementary Figure 20. As for the synchrotron SAXS data shown in Figure 6a, the deviation between model and data at large q is due to the internal porosity and corrugated surface of the assemblies.

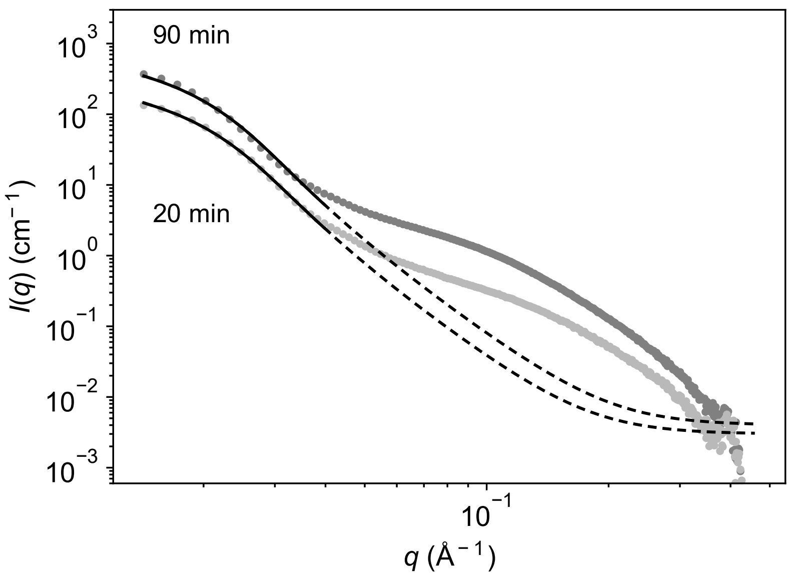

Supplementary Figure 20. Laboratory SAXS data of CoO assemblies after 20 and 90 min reaction time, on absolute scale. The dashed lines correspond to a model of homogeneous spherical particles. The solid lines indicate the fitting range of the least-squares model fit.

We assume the reaction product to be composed of CoO assemblies ($\rho_{\mathrm{particle}} = 51.5 \cdot 10^{-6}\text{Å}^{-2}$) in benzyl alcohol ($\rho_{\mathrm{solvent}} = 9.47 \cdot 10^{-6}\text{Å}^{-2}$) and use the above equation for $I(q)$ for fitting. In the case of absolute scale data and scattering contrast the volume fraction of assemblies is obtained from the model fit. Assuming a mass density of CoO of 6.45 g/cm^3 and a molecular weight of 74.933 g/mol we calculate the final and initial concentration of CoO. The resulting values are given in Supplementary Table 5.

Supplementary Table 5: Volume fractions of assemblies and concentrations of CoO obtained from laboratory SAXS data on absolute scale.

**reaction time**	**20 min**	**90 min**
**volume fraction**	$1.1 \cdot 10^{-4}$	$2.6 \cdot 10^{-4}$
**concentration (mass)**	0.74 mg/ml	1.65 mg/ml
**concentration (amount)**	$9.9 \cdot 10^{-3}$mol/l	$2.2 \cdot 10^{-2}$mol/l

Given an initial concentration of Co of 0.1 mol/l, the final concentration of $2.2 \cdot 10^{-2}$mol/l corresponds to a yield of 22%. The main uncertainty of the concentration measurement is the fit uncertainty of the model, since the data range does not reach the intensity plateau needed to unambiguously determine the particle size distribution. We estimate this uncertainty as 20%, based on a variation of the fitting range.

Supplementary Notes 7. PXRD data analysis

In order to probe for crystallinity, powder X-ray diffraction patterns were recorded on the same samples as the SAXS data (Supplementary Figure 21). The sample at 20 min reaction time shows some very sharp reflections, which presumably come from a crystallized reaction intermediate, and are not observed in the in situ total scattering experiment (Figure 4 and Supplementary Figure 12). A crystalline PXRD signal related to CoO was not observed at this reaction time. Starting from 40 min reaction time, Bragg reflections of cubic CoO were seen. A Scherrer analysis of the widths of the reflections yields crystallite sizes of (4.9 ± 2.8) nm, (5.8 ± 1.0) nm, and (6.0 ± 1.2) nm for 40, 60 and 90 min reaction time, respectively. Details of the Scherrer analysis are given below.

The PXRD reflections of CoO were fitted with a sum of Lorentzian functions of the scattering angle 2ϑ. The widths were then analyzed using the Scherrer equation for the crystallite size d:[17, 18]

$$d = \frac{K\lambda}{\beta \cos\theta}$$

K=1 is a numerical shape factor, λ = 0.71 Å is the X-ray wavelength. θ is the Bragg angle in radians of a reflection of width β.

For β, we use the full width at half maximum (FWHM) B of the fitted reflection, corrected by the instrumental resolution $B_1 \approx 3.3$ mrad. B_1 is the measured angle-dependent FWHM of a LaB_6 powder standard.

In case of a strong, Lorentzian broadening $B_2 = \beta$ due to particle size, and a weak, Gaussian instrumental broadening B_1, the following approximation holds:[19]

$$\frac{B_2}{B} \approx 1 - \frac{\pi}{2}\left(\frac{B_1}{B}\right)^2$$

We solve for $B_2 = \beta$ and obtain

$$\beta \approx \frac{2B^2 - B_1^2\pi}{2B}$$

In Supplementary Table 6, we report the mean and standard deviation of d obtained from 5 reflections (3 for the 40 min data set). The estimate of the number of crystallites is calculated as $N = 0.74 \cdot \frac{D^3}{d^3}$, with the pre-factor assuming dense packing of spherical crystallites in a spherical particle. This number shows an increasing trend as the values obtained from the combination of assembly sizes from SAXS with crystallite sizes from PDF.

Supplementary Table 6: Number of crystallites per particle.

**reaction time**	**40 min**	**60 min**	**90 min**
**D (from SAXS)**	45 nm	52 nm	58 nm
**d (from PXRD)**	(4.9 ± 2.8) nm	(5.8 ± 1.0) nm	(6.0 ± 1.2) nm
**N**	573	533	668

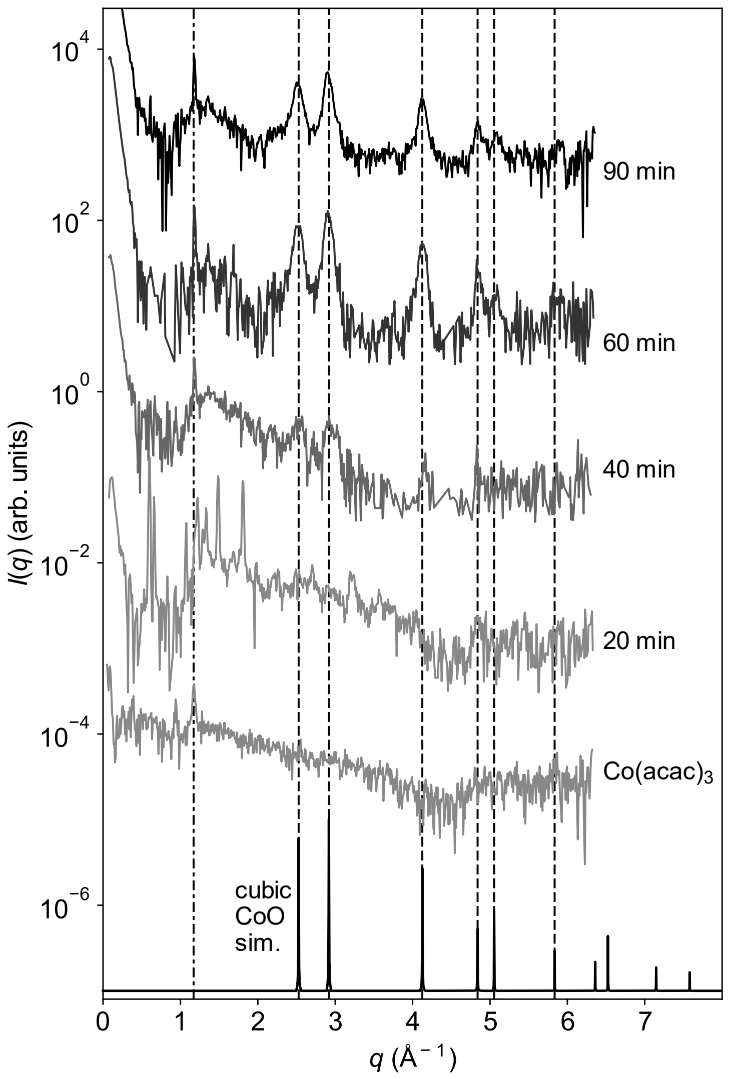

Supplementary Figure 21. PXRD data of dried CoO particles and $Co(acac)_3$ precursor. Positions of the reflections for 40, 60 and 90 min reaction time match cubic CoO ($a = b = c = 4.31$ Å, $\alpha = \beta = \gamma = 90.00°$, dashed lines). Scherrer analysis of the reflection width shows a crystallite size of 4.9 nm, 5.8 nm, and 6.0 nm, for 40, 60, and 90 min, respectively. The $Co(acac)_3$ precursor shows only one clear reflection, which is present also in the reacted samples (dash-dotted line). For clarity, only points with intensity above $10^{-5} sr^{-1}$after background subtraction are shown.

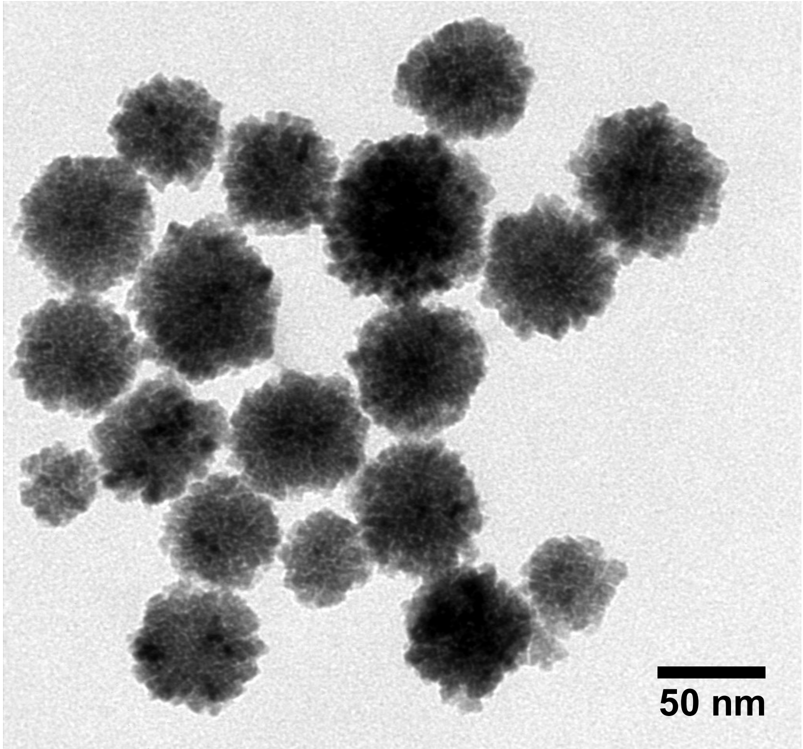

Supplementary Figure 22. TEM image of CoO particles after 90 min reaction time, from the same batch as measured in SAXS. The average particle diameter is 60 nm for the assemblies, and 8-11 nm for the small crystallites.

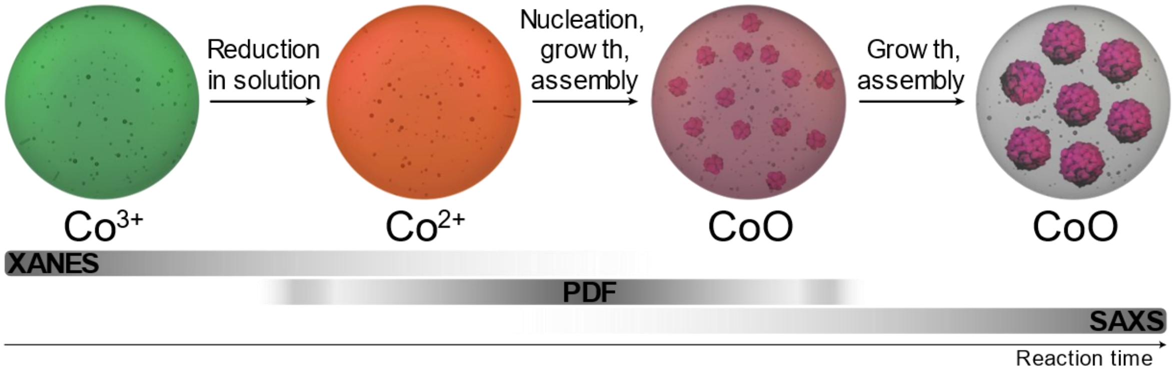

Supplementary Figure 23. Overview of the emergence of nano-assemblies of polyhedrally shaped CoO nanocrystals. We use complementary X-ray spectroscopic and scattering methods to reveal all steps of the non-classical formation pathway.

References

1. Staniuk, M., Hirsch, O., Kränzlin, N., Böhlen, R., van Beek, W., Abdala, P. M. & Koziej, D. Puzzling mechanism behind a simple synthesis of cobalt and cobalt oxide nanoparticles: In situ synchrotron X-ray absorption and diffraction studies. Chemistry of Materials **26**, 2086-2094 (2014).
2. Sasaki, S., Fujino, K. & Tacéuchi, Y. X-ray determination of electron density distributions in oxides, MgO, MnO, CoO, and NiO, and atomic scattering factors of their constituent atoms. Proceedings of the Japan Academy B **55**, 43-48 (1979).
3. de Juan, A., Jaumot, J. & Tauler, R. Multivariate Curve Resolution (MCR). Solving the mixture analysis problem. Analytical Methods **6**, 4964-4976 (2014).
4. Jaumot, J., Gargallo, R., de Juan, A. & Tauler, R. A graphical user-friendly interface for MCR-ALS: a new tool for multivariate curve resolution in MATLAB. Chemometrics and Intelligent Laboratory Systems **76**, 101-110 (2005).
5. Jaumot, J., de Juan, A. & Tauler, R. MCR-ALS GUI 2.0: New features and applications. Chemometrics and Intelligent Laboratory Systems **140**, 1-12 (2015).
6. Windig, W. & Stephenson, D. A. Self-modeling mixture analysis of second-derivative near-infrared spectral data using the simplisma approach. Analytical Chemistry **64**, 2735-2742 (1992).
7. Booth, C. H. & Bridges, F. Improved self-absorption correction for fluorescence measurements of extended x-ray absorption fine-structure. Physica Scripty **T115**, 202-204 (2005).
8. Ludi, B., Suess, M. J., Werner, I. A. & Niederberger, M. Mechanistic aspects of molecular formation and crystallization of zinc oxide nanoparticles in benzyl alcohol. Nanoscale **4**, 1982-1995 (2012).
9. Bilecka, I., Elser, P. & Niederberger, M. Kinetic and thermodynamic aspects in the microwave-assisted synthesis of ZnO nanoparticles in benzyl alcohol. ACS Nano **3**, 467-477 (2009).
10. Rehr, J. J., Kas, J. J., Vila, F. D., Prangebc, M. P. & Jorissena, K. Parameter-free calculations of X-ray spectra with FEFF9. Physical Chemistry Chemical Physics **12**, 5503–5513 (2010).
11. Chen, L., Mashimo, T., Iwamoto, C., Okudera, H., Omurzak, E., Ganapathy, H. S., Ihara, H., Zhang, J., Abdullaeva, Z., Takebe, S. & Yoshiasa, A. Synthesis of novel CoCx@C nanoparticles. Nanotechnology **24**, 045602 (2013).
12. von Chrzanowski, L. S., Lutz, M. & Spek, A. L. alpha-Tris(2,4-pentanedionato-kappa(2)O,O')cobalt(III) at 240, 210, 180, 150 and 110 K. Acta Crystallographica C **63**, m283-m228 (2007).
13. Vreshch, V. D., Yang, J. H., Zhang, H., Filatov, A. S. & Dikarev, E. V. Monomeric square-planar cobalt(II) acetylacetonate: mystery or mistake? Inorganic Chemistry **49**, 8430-8434 (2010).
14. Cotton, F. A. & Elder, R. C. Crystal structure of tetrameric cobalt(II) acetylacetonate. Inorganic Chemistry **4**, 1145-1151 (1965).
15. Radón, M., Srebro, M. & Broclawik, E. Conformational stability and spin states of cobalt(II) acetylacetonate: CASPT2 and DFT study. Journal of Chemical Theory and Computation **5**, 1237–1244 (2009).
16. Kotlarchyk, M., Stephens, R. B. & Huang, J. S. Study of Schultz distribution to model polydlsperslty of microemulsion droplets. The Journal of Physical Chemistry C **92**, 1533-1538 (1988).
17. Saloga, P. E. J. & Thunemann, A. F. Microwave-assisted synthesis of ultrasmall zinc oxide nanoparticles. Langmuir **35**, 12469-12482 (2019).
18. Hargreaves, J. S. J. Some considerations related to the use of the Scherrer equation in powder X-ray diffraction as applied to heterogeneous catalysts. Catalysis, Structure & Reactivity **2**, 33-37 (2016).
19. Ruland, W. The integral width of the convolution of a Gaussian and a Cauchy distribution. Acta Crystallographica C **19**, 581 (1965).
